# Effect of maternal smoking in pregnancy and childhood on child and adolescent sleep outcomes to 21 years: a birth cohort study

**DOI:** 10.1186/s12887-019-1439-1

**Published:** 2019-03-06

**Authors:** Frances O’Callaghan, Michael O’Callaghan, James G. Scott, Jake Najman, Abdullah Al Mamun

**Affiliations:** 1 School of Applied Psychology and Menzies Health Institute, Griffith University, Gold Coast, 4222 Australia; 2 School of Medicine, The University of Queensland, Brisbane, 4101 Australia; 3 Faculty of Medicine, The University of Queensland Centre for Clinical Research and Metro North Mental Health, Royal Brisbane and Women’s Hospital, QLD, Brisbane, 4029 Australia; 4 School of Public Health, The University of Queensland, QLD, Brisbane, 4006 Australia; 5 School of Social Science, The University of Queensland, Brisbane, 4072 Australia

**Keywords:** Sleep, Smoking, Pregnancy, Birth cohort, Childhood, Adolescence, Young adults

## Abstract

**Background:**

The effects of prenatal maternal smoking have been studied extensively, however little research has examined the effects of prenatal exposure to maternal smoking on offspring sleep, particularly over several developmental periods. We examined the effects of prenatal maternal smoking and postnatal smoking from birth to 14 years, on offspring sleep at 6 months, 5, 14 and 21 years.

**Methods:**

This was a prospective, community-based birth cohort study involving 7223 women who delivered a singleton child in Brisbane, Australia between 1981 and 1983. Women were recruited at the first antenatal visit. Offspring sleep problems were reported by mothers at 6 months, 5 and 14 years, and by youth at 14 and 21 years. 3738 mothers prospectively reported their smoking status from pregnancy to 14 years postpartum. Youth snoring was reported by mothers at 14 years and by youth at 21 years. Multinomial logistic regression analyses were performed.

**Results and discussion:**

Prenatal maternal smoking was independently associated with an increased risk of offspring adolescent parasomnias including walking and talking in sleep and nightmares, and an increased likelihood of being in the highest quintile for maternal and youth reported sleep problems at 14 years. Maternal postnatal smoking was associated with increased likelihood of offspring snoring at 14 years.

**Conclusions:**

Exposure to maternal prenatal smoking has different effects on offspring sleep compared to exposure to postnatal smoking. Prenatal smoking exposure may be associated with changes in neurodevelopment whereas postnatal smoking is more likely to affect the respiratory system. These findings highlight the long lasting and potentially serious clinical effects of exposure to pre and postnatal maternal smoking on offspring, the mechanisms by which warrant further investigation.

**Electronic supplementary material:**

The online version of this article (10.1186/s12887-019-1439-1) contains supplementary material, which is available to authorized users.

## Background

Sleep is related to various aspects of mental, cognitive and physical wellbeing in children and adults [1]. There is abundant evidence of the adverse consequences of sleep problems on individuals including attention problems [2], neuropsychological problems [3], learning [4], emotional and behavioural problems [5, 6], daytime functioning and quality of life [7, 8]. The effects of maternal smoking during pregnancy have also been studied extensively, with exposed children being at greater risk of a range of adverse outcomes [9–13], some evident even in adulthood, for example nicotine dependence [14] and adverse effects on intelligence [15]. More recently studies have suggested an association between prenatal maternal smoking and sleep problems in children [16–19]. If confirmed and the relationship persisted, this could potentially be an important mechanism contributing to adolescent and young adult sleep problems and their associated morbidity.

There are several mechanisms by which prenatal exposure to smoking may explain subsequent adverse outcomes for the child. These include direct effects of tobacco products, including nicotine, on the developing brain’s acetylcholine neurotransmitter systems and cells involved in sleep regulation [20–22]; known effects on later child behaviour; and confounding by family and social factors. A recent review of epidemiological and animal studies concluded that the pathophysiology for this diverse spectrum of outcomes remains incompletely understood [23] though epigenetic processes may be influential [24].

Few studies, however, have examined the effects of prenatal exposure to smoking on offspring sleep. A polysomnographic study in preterm infants demonstrated disrupted sleep structure and continuity and increased movement in infants exposed to in utero maternal smoking [25]. Two recent studies have examined these associations longitudinally, both indicating that the effects are not explained by postnatal maternal smoking. In one of these studies, prenatal nicotine exposure in a sample of 139 children was associated with sleep problems to 9 years [26]. A second study of low SES, high-risk mothers recruited after delivery found a dose-response relationship between prenatal tobacco exposure and persisting sleep problems in 808 children from 1 month to 12 years [27]. These studies provide valuable information and raise two important questions: (1) Are these effects evident in children who are more representative of the general community? (2) To what extent do sleep problems associated with prenatal nicotine exposure persist into adolescence and adulthood?

The current paper examines the association between prenatal and postnatal maternal smoking, and offspring sleep problems in a large longitudinal community birth cohort study. We aimed to examine if exposure to prenatal maternal smoking increased the risk of sleep problems and snoring in offspring from 5 to 21 years, comparing this to the effect of postnatal maternal smoking on offspring sleep problems.

## Methods

### Study design and population

The Mater-University of Queensland Study of Pregnancy (MUSP) comprises a birth cohort of 7223 singleton infants born during 1981–83, with mothers being enrolled at the first antenatal visit (average 18 weeks gestation) [28]. Questionnaires were completed by mothers at enrolment, delivery, 6 months, and 5 years and by both mothers and offspring at 14 and 21 years (Additional files 1, 2, 3, 4, 5, 6, and 7). Numbers vary depending on the follow-up stage and sleep items.

### Standard protocol approvals, registrations, and patient consent

The study was approved by the Human Research Ethics Committees of the Mater Hospital and The University of Queensland, with written informed consent being obtained from the mother at each stage of follow-up and from the youth at 21 years.

### Maternal smoking status

Maternal smoking pattern over the previous week was examined in maternal questionnaires at the first clinic visit (FCV) (average 18 weeks gestation), 3–5 days after delivery, 6 months, 5 years and 14 years. Mothers were asked to record whether they smoked (yes or no) and if yes, their frequency and quantity of smoking in the previous week. Mothers were also asked at the FCV about whether and how much they smoked before they became pregnant. At 3–5 days after delivery, mothers were asked to recall their smoking level during the last trimester.

Information on maternal smoking was gathered in circumstances designed to maximize the accuracy of the data. This involved interviews conducted within a clinical setting, assurances of confidentiality, detailed questions, and trained interviewers. Reports do vary as to the accuracy of self-reported smoking among pregnant women. Significant agreement between self-reported smoking and serum cotinine levels (the major metabolite of nicotine) has been found [29, 30]. However, varying levels of under-reporting of smoking by pregnant women have been noted [31, 32] and it is important to acknowledge the importance of the setting in which information is collected. This is highlighted by Carabello and colleagues [33] whose findings from a population-based survey of adults regarding their smoking status attest to the accuracy of self-reported smoking status if collected in a private medial setting. Pickett and colleagues [34] illustrate the complex nature of this issue, as their prospective research of pregnant women involved repeated measures of both self-reported smoking status and that based on cotinine levels. They concluded that in epidemiological studies where the intensity and timing of exposure is of particular interest, self-reported smoking status provides a valid measure of fetal exposure. The information was collected in the early 1980’s when the prevalence and extent of smoking were higher and the issue was less prominent as a public health concern. Any potential bias is likely to lead to underreporting of cigarette use, with reduction of effect size.

### Prenatal smoking

The category of prenatal smoking included mothers who reported smoking in early or late pregnancy and other times. Maternal smoking status was categorised as ‘never smoked at any stage of the study’ and ‘smoked during pregnancy’ (i.e. smoked in either early or late pregnancy and other times).

### Postnatal smoking

The final category consisted of women who smoked postnatally but not during or before pregnancy (women who responded “no” to smoking before pregnancy, at FCV and third trimester but “yes” at 6-months, 5 years or 14 years). The offspring whose mothers smoked during pregnancy and other times were exposed to the effects of maternal smoking whilst in utero as well as the effects of maternal smoking during childhood whereas the offspring of mothers who smoked postnatally but not during or before pregnancy were exposed to passive maternal smoking in infancy/childhood only. This enabled examination of the effects of in utero exposure to smoking over and above the effects of exposure to maternal smoking during early post-natal and childhood development. These categories are mutually exclusive. Women who smoked only before pregnancy were excluded.

### Offspring sleep problems

At 5 years, mothers were asked how often their child had experienced trouble sleeping over the previous year, rated as often, sometimes, and never/rarely. At 14 years, mothers completed the Child Behaviour Checklist and the youth completed the Youth Self Report [35]. These assessments were chosen because they cover broad domains of mental health and child behavioural issues, they have established reliability and validity [35–37] and they have been widely used across many countries. The YSR is a standardized self-report questionnaire for adolescents aged from 11 to 18 years. The CBCL is a maternal report questionnaire that assesses the same behavioural subscales as the YSR [38]. Both scales contain the same five common items examining different aspects of sleep initiation and maintenance, parasomnias, and daytime tiredness over the previous 6 months. These five items were “trouble sleeping”, “sleeps less than most kids”, “nightmares”, “sleeps more than most kids”, and “overtired”, with each item rated as often, sometimes, and rarely/never. “Talks or walks in sleep” was an additional question in the maternal CBCL. Mothers were also asked if their child had snored over the previous year. At 21 years many, though not all, of the items of the Pittsburgh sleep inventory [39] were administered to the young adult, as well as additional sleep questionnaire items. Four questions examined difficulties in initiating or maintaining sleep (trouble sleeping, sleep quality, restless sleep and night waking) and three questions examined daytime somnolence (trouble staying awake, day time drowsiness and overtired). Young adults also reported nightmares and snoring. Presence of problems was rated over the previous month.

We created composite indicators of sleep problems at 14 and 21 years, separately for maternal reported sleep items, youth reported sleep items and young adult reported sleep items. For sleep items (e.g. snoring) with a response option of ‘yes’, a score of ‘1’ was assigned, and ‘0’ for a response of ‘no’. Other items, with response options in three categories, were assigned ‘0’ for ‘rarely/never’ or equivalent response, ‘1’ for ‘sometimes’ or equivalent response, and ‘2’ otherwise. All the items were summed after this scoring. The overall score was grouped into three categories as follows: ‘lower 20%’, ‘middle 20–80%’, and upper 20%, with upper 20% having the highest number of sleep symptoms.

### Confounders and mediators

Two groups of factors were examined: These were (i) other pregnancy lifestyle exposures and (ii) social and maternal factors. (i) Other pregnancy exposures examined were alcohol, tea and coffee. At the first pregnancy visit and within 1–3 days of birth, questionnaire items examined amount and frequency of alcohol intake (classified as nil, <one glass a day and > one glass a day). Few mothers drank heavily. Coffee and tea each had four categories of none, 1, 2–3 or ≥ 4 cups per day. Analyses were undertaken combining tea and coffee consumption and also examining their effect on offspring sleep separately. Only 2% of mothers reported using marijuana in pregnancy and analysing the data with and without this group produced no differences in the results. (ii) Social and demographic factors were maternal age and level of education, and family income at the FCV. The cut-off for low income levels was the lowest approximate third, depending upon the distribution. At the first antenatal visit mothers were asked four questions concerning whether the pregnancy was planned or wanted and classified as planned/wanted, unplanned/unwanted. Duration of breastfeeding was reported at the six-month follow-up and classified as nil, < 4 months or > 4 months.

### Statistical analyses

The relationship between the maternal smoking variable (3 categories) and the 22 sleep measures at different follow-up phases (refer Table 2) was initially examined, with the chi squared test being administered for statistical significance. As each test reflected the initial study hypothesis, a two tailed *P*-value of < 0.05 was taken to indicate initial statistical significance. However, due to multiple statistical comparisons, the effect of applying the Bonferroni correction was also examined. The relationship between maternal smoking and the eight sleep questions significant in this initial analysis is described in Table 2. To increase the statistical precision, for some outcomes we combined ‘sometimes’ and ‘often’ responses as one category (see Table 3) for the multivariable analyses. A sensitivity analysis was conducted to examine these results further. Of women who smoked cigarettes in pregnancy, more than half of them reported smoking ≥10 cigarettes daily. No dose-response relationship was found. Additionally, as some of the cell frequencies were small, especially for the postnatal only smoking group, in the adjusted analyses we considered coffee, tea, alcohol in pregnancy, maternal age and education. Combining tea and coffee or considering them separately did not have any impact on the results. In the sensitivity analysis, we added other confounding factors and found the effect size remained consistent. An adjusted analysis was performed using multinomial logistic regression examining sequentially the effects of other pregnancy lifestyle exposures, social factors and maternal factors, with all factors included in a final fully adjusted model. In these multinomial logistic models, the groups of prenatal exposure, and postnatal exposure, were contrasted to never smoking mothers as the reference category.

## Results

### Descriptive analyses

Table 1 describes the total birth cohort and the study group at each stage of follow-up. Mothers of children lost to follow-up were more likely to be younger, less well educated, more financially disadvantaged and the children more likely to be of birthweight < 2500 g or gestation < 37 weeks. Of 3954 women who prospectively reported their smoking status from pregnancy to 14 years follow-up, 55% never smoked, 41% smoked at some stage of pregnancy and other time and 4% smoked after pregnancy but not during or before pregnancy.

**Table 1:** Comparison of birth cohort and study group at each stage of follow-up

Variable	Birth cohort^a^ (*n* = 7223) *n*%		5 years (*n* = 4249) %	14 years (*n* = 4155) %	21 years (*n* = 2913) %
Maternal age (yrs)					
15–19	1184	16.4	12.4	12.5	11.4
20–35	5726	79.3	83.2	83.2	83.8
> 35	313	4.3	4.4	4.3	4.7
Level maternal education					
Incomplete high	1305	18.2	16.3	16.0	15.2
Complete high	4609	64.3	64.4	64.6	64.3
Post high	1256	17.5	19.4	19.4	20.5
Family income (antenatal)					
> $10,400	4441	65.8	71.3	71.6	72.3
< $10,400	2308	34.2	28.7	28.4	27.7
Maternal depression					
No	6262	88.4	90.7	90.8	91.7
Yes	823	11.6	9.3	9.2	8.3
Gender					
Male	3758	52.0	52.1	52.1	47.5
Female	3465	48.0	47.9	47.9	52.5
Birth weight					
> 2500 g	6911	95.7	96.2	96.2	96.5
< 2500 g	311	4.3	3.8	3.8	3.5
Gestation					
> 37 wks	6927	95.9	96.1	96.1	96.1
< 37 wks	296	4.1	3.9	3.9	3.9

^a^Numbers vary slightly due to missing data

### Bivariate association between maternal smoking status and offspring sleeping

Table 2 provides an overview of the 22 sleep questions ordered according to their follow-up phase, their distribution, and the level of statistical significance for their overall association with maternal smoking status during and after pregnancy. Eight of the sleep measures were initially significant at *P* < 0.05, five of these at 14 years and three at 21 years. At 14 years the significant associations were: mother reports of talking or walking in sleep (*P* < 0.001), sleep more than other kids (*P* = 0.044) and snoring over the last year (*P* = 0.001). Youth-reported nightmares at 14 years was associated with maternal smoking in utero (*P* = 0.01). At 21 years, young adult-reported nightmares (*P* = 0.01) and being restless and trouble staying awake p (*P =* 0.017) were significant. There was no association between troubles sleeping at five years and maternal smoking. When the Bonferroni test was applied to the initial 24 comparisons, walking and talking in sleep retained significance. Each of the eight overall sleep measures was further examined for an interaction between smoking and gender and no interaction terms were significant.

**Table 2:** Distribution of sleep measures at 6-months, 5, 14 and 21 years and overall significance of relationship to smoking (never, smoked postnatally but not in pregnancy and pre-pregnancy, pregnancy+other times)

		Maternal Smoking			
	*n* (%)	Never (*n* = 2174)%	Smoked postnatally but not in pregnancy and pre-pregnancy (*n* = 170)%	Smoked in pregnancy + other times (*n* = 1610) %	*P*
6-months					
Sleeplessness					
Almost everyday	282 (7.2)	145 (6.8)	6 (3.6)	131 (8.3)	
Few times a week	419 (10.7)	213 (9.9)	20 (11.9)	186 (11.7)	
Few times a month	458 (11.7)	264 (12.3)	19 (11.3)	175 (11.0)	
Few times Rarely/never	2744 (70.3)	1527 (71.1)	113 (73.2)	1094 (68.9)	0.083
5-years (past year)					
Trouble sleeping					
often	108 (2.7)	52 (2.4)	4 (2.4)	52 (3.3)	
sometimes	839 (21.3)	453 (20.9)	31 (18.2)	355 (22.2)	
rarely/never	2996 (76.0)	1666 (76.7)	135 (79.4)	1195 (74.6)	0.302
14 years (mother)					
Trouble sleeping					
often	69 (1.7)	40 (1.8)	4 (2.3)	25 (1.6)	
sometimes	513 (12.9)	272 (12.5)	29 (17.0)	212 (13.1)	
rarely/never	387 (85.3)	1873 (85.7)	138 (80.7)	1376 (85.30)	0.426
Sleeps less					
often	92 (2.3)	48 (2.2)	6 (3.5)	38 (2.4)	
sometimes	379 (9.5)	183 (8.4)	23 (13.5)	173 (10.7)	
rarely/never	3503 (88.2)	1956 (89.4)	142 (83.0)	1405 (86.9)	0.032
Nightmares					
often	35 (0.9)	15 (0.7)	4 (2.4)	16 (1.0)	
sometimes	592 (14.9)	306 (14.0)	26 (15.3)	260 (16.1)	
rarely/never	3341 (84.2)	1865 (85.3)	140 (82.3)	1336 (82.9)	0.063
Talks/walks					
often	133 (3.4)	49 (2.2)	8 (4.7)	76 (4.7)	
sometimes	952 (24.0)	498 (22.8)	37 (21.6)	417 (25.9)	
rarely/never	2881 (72.6)	1636 (74.9)	126 (73.7)	1119 (69.4)	< 0.001
Sleeps more					
often	96 (2.4)	54 (2.5)	2 (1.2)	40 (2.5)	
sometimes	572 (14.4)	282 (12.9)	28 (16.4)	262 (16.2)	0.044
rarely/never	3300 (83.2)	1845 (84.6)	141 (82.5)	1314 (81.3)	
Overtired					
often	134 (3.4)	76 (3.5)	5 (3.0)	53 (3.3)	
sometimes	1630 (41.2)	897 (41.1)	80 (47.1)	653 (40.6)	
rarely/never	2197 (55.5)	1210 (55.4)	85 (50.0)	902 (56.1)	0.594
Snoring					
Yes	932 (23.5)	463 (21.3)	53 (30.8)	416 (25.8)	
No	3032 (76.5)	1715 (78.7)	119 (69.2)	1198 (74.2)	< 0.001
14 years (youth)					
Trouble sleeping					
often	216 (5.8)	127 (6.1)	7 (4.2)	82 (5.5)	
sometimes	1276 (34.1)	681 (32.6)	61 (36.8)	534 (36.1)	
rarely/never	2246 (60.1)	1284 (61.4)	98 (59.0)	864 (58.4)	0.200
Sleep less					
often	304 (7.7)	152 (7.0)	13 (7.6)	139 (8.6)	
sometimes	1199 (30.3)	662 (30.5)	51 (30.2)	486 (30.2)	
rarely/never	2450 (62.0)	1356 (62.5)	107 (62.6)	987 (61.2)	0.485
Nightmares					
often	107 (2.7)	46 (2.1)	6 (3.5)	55 (3.4)	
sometimes	971 (24.6)	502 (23.1)	43 (25.3)	426 (26.5)	
never	2876 (72.7)	1626 (74.8)	121 (71.2)	1129 (70.1)	0.010
Sleeps more					
often	282 (7.1)	144 (6.7)	8 (4.7)	130 (8.1)	
sometimes	1183 (30.0)	664 (30.7)	50 (29.2)	469 (29.1)	
rarely/never	2484 (62.9)	1358 (62.7)	113 (66.1)	1013 (62.8)	0.269
Overtired					
often	380 (9.6)	20 (9.5)	11 (6.5)	164 (10.2)	
sometimes	1983 (50.3)	1089 (50.3)	94 (55.3)	800 (49.8)	
rarely/never	1578 (40.0)	872 (40.3)	65 (38.2)	641 (39.9)	0.482
21 years		(*n* = 1611)	(*n* = 100)	(*n* = 1081)	
Trouble sleeping					
often	366 (13.1)	192 (11.9)	13 (13.0)	161 (14.9)	
somewhat	1090 (38.0)	635 (39.4)	39 (39.0)	416 (38.5)	
not true	1336 (47.9)	784 (48.7)	48 (48.0)	504 (46.6)	0.279
Nightmares					
often	110 (3.9)	56 (3.5)	3 (3.0)	51 (4.7)	
somewhat	592 (21.2)	320 (19.9)	11 (11.0)	261 (24.1)	
not true	2089 (74.9)	1234 (6.7)	86 (86.0)	769 (71.1)	0.001
Overtired					
often	387 (13.9)	220 (13.7)	15 (15.3)	152 (14.0)	
somewhat	1310 (47.0)	761 (47.5)	43 (43.9)	506 (46.5)	
not true	1093 (39.2)	622 (38.8)	40 (40.8)	431 (39.6)	0.952
Restless sleep					
Three+ per week	444 (16.0)	238 (15.0)	15 (15.0)	191 (17.7)	
Once or twice/week	940 (34.0)	534 (33.6)	24 (24.0)	382 (35.4)	
< once/week	1385 (50.0)	819 (51.5)	61 (61.0)	505 (46.9)	0.017
Night walking					
Three+ per week	541 (19.6)	303 (19.0)	13 (13.4)	225 (20.9)	
Once or twice/week	612 (22.1)	343 (21.6)	22 (23.7)	247 (22.9)	
< once/week	1613 (58.3)	946 (59.4)	62 (63.9)	605 (56.2)	0.252
Drowsy at daytime					
Three+ per week	636 (22.9)	363 (22.8)	16 (16.2)	257 (23.8)	
Once or twice/week	1136 (40.9)	642 (40.3)	42 (42.4)	452 (41.8)	
< once/week	1003 (36.1)	590 (37.0)	41 (41.4)	372 (34.4)	0.314
Trouble staying awake					
At least once/week	229 (8.6)	140 (8.7)	4 (4.0)	85 (7.8)	
< once/week	621 (22.2)	386 (23.9)	21 (20.8)	214 (19.6)	
No	1954 (69.7)	1087 (69.4)	76 (75.3)	791 (72.6)	0.044
Sleep quality					
Largely bad	82 (2.9)	38 (2.4)	3 (3.2)	41 (3.8)	
Fairly bad	452 (16.1)	257 (16.0)	11 (10.9)	184 (16.9)	
Fairly/very good	2268 (81.0)	1315 (81.7)	87 (86.1)	866 (79.4)	0.226
Snoring					
Three+/week	183 (6.7)	100 (6.4)	3 (3.1)	80 (7.5)	
Once to twice/week	232 (8.5)	126 (8.0)	8 (8.3	98 (9.2)	
< Once/week	2319 (84.8)	1344 (85.6)	86 (88.7	889 (83.3)	0.316

### Multivariable analyses

In Table 3, the strength of relationship between three mutually exclusive categories of maternal smoking status (no smoking as the reference category) and the eight sleeping outcomes are shown adjusted for other pregnancy exposures, and maternal and social factors. For maternal smoking during pregnancy and other times, mother-reported talks/walks in sleep and youth-reported nightmares were more likely (OR and 95% CI: 1.23, 1.04–1.46 and 1.23, 1.03–1.46 respectively) whereas problems staying awake at 21 years was less likely (OR and 95% CI: 0.80, 0.65–0.98). For mothers who smoked postnatally but not before or during pregnancy, maternal reported offspring snoring at 14 years was more likely (OR and 95% CI: 1.53, 1.06–2.23). The composite sleep variables in Table 4 show that the top 20% and middle 20–80% of maternal reports of sleep problems at 14 years were more likely in the offspring of both maternal smoking groups in the adjusted analysis (OR and 95% CI: 1.26, 1.05–1.50 and 1.36, 1.05, 1.76, respectively). For youth-reported composite sleep problems at 14 years, the only significant association in the adjusted analysis is with smoking in pregnancy and at other times (OR and 95% CI: 1.29, 1.02–1.64). At 21 years, no associations are significant in the adjusted analysis.

**Table 3:** Odds of sleeping problems for offspring at 14 and 21 years by maternal smoking status

		Maternal Smoking			Maternal Smoking		
Sleep	N	Never	Smoked postnatally but not in pregnancy and pre-pregnancy OR (95% CI)	Smoked in pregnancy + other times OR (95% CI)	Never	Smoked postnatally but not in pregnancy and pre-pregnancy OR (95% CI)	Smoked in pregnancy + other times OR (95% CI)
14 y- talks/walks sleep (mothers)			Unadjusted			Adjusted	
Often/Sometimes	944	1.00	1.10 (0.75, 1.60)	**1.38 (1.19, 1.61)**	1.00	1.00(0.67, 1.46)	**1.23 (1.04, 1.46)**
Rarely/never	2514	1.00	Reference	Reference	1.00	Reference	Reference
Snoring last year (mothers)							
Yes	804	1.00	**1.63 (1.13, 2.36)**	**1.28 (1.08, 1.50)**	1.00	**1.53 (1.06, 2.23)**	1.15 (0.96, 1.38)
No	2654	1.00	Reference	Reference	1.00	Reference	Reference
Sleeps more than most kids day or night (mothers)							
Often / Sometimes	584	1.00	1.26 (0.82, 1.94)	**1.26 (1.05, 1.51)**	1.00	1.16 (0.75, 1.80)	1.10 (0.89, 1.34)
Rarely/never	2889		Reference	Reference	1.00	Reference	Reference
Sleep less than most kids day or night (mothers)							
Often/Sometimes	409	1.00	1.56 (0.98, 2.49)	**1.32 (1.07, 1.63)**	1.00	**1.58 (0.98, 2.54)**	1.24 (0.97, 1.56)
Rarely/never	3070	1.00	Reference	Reference	1.00	Reference	Reference
Nightmares (youth)							
Often/sometimes	939	1.00	1.23(0.98, 1.54)	**1.31(1.12,1.52)**	1.00	1.22(0.84, 1.77)	**1.23(1.03,1.46)**
Rarely/never	2502	1.00	Reference	Reference		Reference	Reference
21 years- nightmares (young adult)							
Yes (somewhat, sometimes or very-often)	607	1.00	**0.53 (0.29, 0.99)**	1**.27 (1.05, 1.53)**	1.00	**0.50 (0.26, 0.93)**	1.14 (0.92, 1.41)
No	1820	1.00	Reference	Reference	1.00	Reference	Reference
21 years- restlessness in sleep (young adult)							
Three+ per week	382	1.00	0.91 (0.50, 1.67)	**1.27 (1.00, 1.61)**	1.00	0.80 (0.42, 1.48)	1.03 (0.78, 1.34)
Once or twice/week	820	1.00	0.51 (0.30, 0.88)	1.14 (0.95, 1.37)	1.00	0.49 (0.28, 0.84)	1.08 (0.88, 1.33)
< once /week	1201	1.00	Reference	Reference		Reference	Reference
Trouble staying awake							
No	1702	1.00	Reference	Reference	1.00	Reference	Reference
Yes (<once a week to at least once a week)	748	1.00	0.66 (0.40, 1.10)	**0.78 (0.65, 0.93)**	1.00	0.65 (0.39, 1.08)	**0.80 (0.65, 0.98)**

^a^Adjusted for coffee, tea, alcohol in late pregnancy, maternal age, maternal education, family income, planning of pregnancy and breastfeeding

Odds ratios in bold are significantly different to those of the reference category (*P*<.05)

**Table 4:** Unadjusted and adjusted odds of composite sleeping problems for offspring at 14 and 21 years by maternal smoking status

Composite indicator of sleep problems		Maternal Smoking			Maternal Smoking		
14 years maternal report of sleep	N	Never	Smoked postnatal but not in pregnancy and pre-pregnancy OR (95% CI)	Smoked in pregnancy + other times OR (95% CI)	Never	Smoked postnatal but not in pregnancy and pre-pregnancy OR (95% CI)	Smoked in pregnancy + other times OR (95% CI)
			Unadjusted			Adjusted^a^	
Lower 20%	959 (28.03)	1.00	Reference	Reference	1.00	Reference	Reference
Middle 20–80%	2000 (58.50)	1.00	1.14 (0.77, 1.70)	**1.27 (1.08, 1.49)**	1.00	1.13 (0.75, 1.68)	**1.26 (1.05, 1.50)**
Top 20%	462 (13.50)	1.00	**1.85 (1.11, 3.11)**	**1.63 (1.30, 2.05)**	1.00	1.71 (1.00, 2.89)	**1.36 (1.05, 1.76)**
14 years youth report of sleep							
Lower 20%	854 (25.08)	1.00	Reference	Reference	1.00	Reference	Reference
Middle 20–80%	1924 (56.51)	1.00	0.73 (0.50, 1.09)	1.11 (0.94, 1.31)	1.00	0.74 (0.50, 1.10)	1.13 (0.94, 1.36)
Top 20%	627 (18.41)	1.00	0.97 (0.60, 1.59)	**1.29 (1.05, 1.60)**	100	0.95 (0.58, 1.57)	**1.29 (1.02, 1.64)**
21 years report of sleep							
Lower 20%	745 (31.91)	1.00	Reference	Reference	1.00	Reference	Reference
Middle 20–80%	1193 (51.09)	1.00	0.78 (0.48, 1.26)	1.15 (0.95, 1.40)	1.00	0.74 (0.45, 1.22)	1.10 (0.89, 1.37)
Top 20%	397 (17.00)	1.00	0.69 (0.34, 1.40)	1.23 (0.96, 1.59)	1.00	0.58 (0.28, 1.18)	1.06 (0.80, 1.42)

^a^Adjusted for coffee, tea, alcohol in late pregnancy, maternal age, maternal education, family income, planning of pregnancy and breastfeeding

Odds ratios in bold are significantly different to those of the reference category (*P*<.05)

## Discussion

Compared to the offspring of mothers who were non-smokers on all occasions, those exposed to maternal smoking were more likely to have changes in 5 individual offspring sleep items after adjusting for confounders, 4 of these at 14 years and one item at 21 years. Offspring exposed to maternal smoking whilst in utero were more likely at 14 years to have parasomnias as evidenced by maternal-reported ‘talks and walks in sleep’ and youth-reported nightmares. Offspring snoring and sleeping less at 14 years were associated with mothers who did not smoke in pregnancy though smoked at other times. At 21 years, offspring of mothers who smoked in pregnancy and at other times were less likely to report difficulties staying awake. For the composite sleep measures at 14 years, offspring exposed to smoking in pregnancy and at other times were more likely to be in the highest quintile for sleep problems as reported by both youth and their mothers. According to maternal report, offspring exposed to smoking in the postnatal period only and not during pregnancy were also more likely to be in the highest quintile for sleep problems.

It is noteworthy that the parasomnias (walking and talking in sleep and nightmares) occurred in offspring whose mothers smoked during pregnancy and at other times but were not evident in the offspring who were not exposed to smoking during pregnancy. Stone et al’s [27] prospective longitudinal study of children from a high risk sample with multiple exposures found that postnatal smoking did not contribute significantly to the explanation of a composite measure of sleep problems across the first 12 years. Similarly, in our study, the composite sleep measure showed that offspring exposed to maternal smoking in utero were more likely to have sleep problems at 14 years as reported by both the youth and their mothers.

Prenatal smoking is known to be associated with later behaviour problems in children [9, 40] that may potentially influence parasomnias. The findings for walks/talks in sleep, though not nightmares, were independent of mental health at 14 years measured by the YSR. Walking and talking in sleep are parasomnias that occur in non-REM sleep and reflect transitions from deep to light sleep. The literature suggests that walking in sleep decreases after adolescence to adult levels of up to 4% [41, 42]. Although prevalence decreases with age, the potential for serious injury and aggression involving the individual and others [43], as well as the implications for daytime functioning deficits due to poorer sleep quality suggests that this warrants further investigation. This association may be casual and reflect mechanisms discussed earlier including epigenetic effects of nicotine on the developing neurotransmitter systems. Sleepwalking is increased in Parkinson’s disease where an imbalance of neurotransmitters exists due to loss of dopamine producing neurons [1]. A genetic predisposition [2] is also possible. Our study supports that exposure to maternal smoking in utero but not postnatally may result in neurodevelopmental changes, of which one manifestation is an increased risk of parasomnias.

The lack of significant findings for trouble sleeping at 5 years may reflect the limitation and imprecision of using a one-item measure given their inconsistency with previous research [26, 27]. However, this study cohort differs in not being high risk. At 14 and 21 years, several sleep measures were evaluated. Although these were self or maternal report single-item measures, they included a question on trouble sleeping that Gregory et al. [44] reported to be associated with results from more formal sleep studies. Within this cohort, trouble sleeping behaviours have previously been found to be associated with sleep problems persisting from a young age to adulthood [45].

### Strengths and limitations

Several features of the current study make the results noteworthy. This is the first longitudinal study to examine sleep problems associated with prenatal maternal smoking and postnatal smoking across several developmental periods in a large community sample. This study is best able to examine consequences of prenatal smoking on sleep in adolescence and young adulthood and control for exposure to parental smoking during childhood. The only previous longitudinal research examined outcomes up to 12 years in a very different group – a high risk cohort with pregnancy exposures [27]. The analyses adjusted for a number of psychosocial characteristics that could potentially confound any relationship between maternal smoking and offspring sleep. The strategy for data analysis used in our study also has advantages for exposures such as cigarette use that are strongly correlated over time. Although we could examine the effect of maternal smoking during pregnancy on offspring sleep separately from smoking at other times, a limitation of this study is that level of smoking could not be evaluated using this methodology so a threshold effect with heavy prenatal smoking cannot be excluded. A further limitation is the lack of information on exposure to other sources of passive smoking including smoking by fathers. The exposure to second hand smoking by other people in the household may impact on the respiratory systems of the offspring resulting in increased risk of parasomnias and snoring. Smoking by others in the home, however, is likely to be correlated with maternal smoking and disentangling what is a direct effect of maternal smoking and a result of passive smoking was not possible in this study. While maternal smoking was measured only with self-reports, these involved detailed questions asked by trained interviewers in a confidential, clinical setting. There is therefore a possibility of a social desirability bias leading to under-reporting of smoking status.

Other limitations include the use of questionnaire measures to assess sleep and the level of attrition. We have previously assessed the impact of attrition using multiple imputation and sensitivity analyses, with little impact on the findings [28]. Comparisons to other cohort studies have also yielded similar results [46]. Attrition was more likely in those offspring whose mothers had adverse social circumstances and adjustment for these in our analysis made little difference to the findings. Moreover, the present results would only be biased if the associations were in the opposite direction in participants not assessed at each phase of the research which would be unlikely.

## Conclusions

The association between prenatal maternal smoking and adolescent offspring parasomnias of walking in sleep and talking in sleep at 14 years have important clinical implications due to the potential for serious injury and aggression, as well as consequences for sleep quality and daytime functioning, as noted previously. This study supports the research of others that exposure to maternal smoking in utero may affect neurodevelopment which has an impact on offspring even in the adolescent years. This is more evidence of the serious adverse effects of smoking which persist well beyond those direct effects on the individual and are associated with changes in other family members in systems of the body, the mechanisms by which warrant further investigation.

## Additional files

Additional file 1: MUSP maternal first clinic visit. (PDF 304 kb)

Additional file 2: MUSP obstetric. (PDF 313 kb)

Additional file 3: MUSP maternal 6 months. (PDF 326 kb)

Additional file 4: MUSP maternal 5 years. (PDF 294 kb)

Additional file 5: MUSP maternal 14 years. (PDF 532 kb)

Additional file 6: MUSP youth 14 years. (PDF 143 kb)

Additional file 7: MUSP youth 21 years. (PDF 968 kb)

Additional file 8: MUSP input data 2019. (XLSX 568 kb)

Additional file 9: MUSP smoking and sleep input data codebook. (PDF 354 kb)

## Abbreviations

CBCL: Child behaviour checklist
CI: Confidence interval
MUSP: Mater – University of Queensland Study of Pregnancy
OR: Odds ratio
YSR: Youth self report
