# Supplementary material for: Effect of maternal smoking in pregnancy and childhood on child and adolescent sleep outcomes to 21 years: a birth cohort study
Source: BMC Pediatr. 2019 Mar 6;19:70. doi: 10.1186/s12887-019-1439-1 (PMC6402153; doi:10.1186/s12887-019-1439-1)
Supplement: Supplementary file 1 — MUSP maternal first clinic visit. (PDF 304 kb) [file 12887_2019_1439_MOESM1_ESM.pdf]

MUSP Maternal First Clinic Visit

Phase: 1

Code No. ________________

Date: ________________

First we would like to know how you felt when you found out you were pregnant. How well do the following statements describe how you felt when you found out you were pregnant.

PLEASE CIRCLE ONE NUMBER FOR EACH STATEMENT.

	Strongly Agree	Agree	Unsure - Mixed Feelings	Disagree	Strongly Disagree
1. I felt overjoyed	1	2	3	4	5
2. I would have preferred not to become pregnant	1	2	3	4	5
3. I felt unhappy	1	2	3	4	5
4. I felt it was the worst thing that could have happened to me	1	2	3	4	5

How well do the following describe how you feel now.

5. Pregnancy makes me feel feminine	1	2	3	4	5
6. I have to give up a lot for this pregnancy	1	2	3	4	5
7. Pregnant women are particularly attractive	1	2	3	4	5
8. Being pregnant is far too restricting	1	2	3	4	5

We are also interested in how your partner felt when he first knew you were pregnant.
Even if you haven't discussed it with him answer the following questions according to how you think he felt.

PLEASE CIRCLE ONE NUMBER FOR EACH STATEMENT FOR QUESTIONS 9 - 12.

		No Partner	Strongly Agree	Agree	Unsure - Mixed Feelings	Disagree	Strongly Disagree
9.	I think he felt overjoyed	1	2	3	4	5	6
10.	I think he would have preferred if I hadn't become pregnant at this time	1	2	3	4	5	6
11.	I think he felt unhappy	1	2	3	4	5	6
12.	I think he felt it was the worst thing that could have happened	1	2	3	4	5	6

Below is a list of statements about this pregnancy which may be relevant to you.

PLEASE CIRCLE ONE NUMBER FOR EACH STATEMENT FOR QUESTIONS 13 - 17.

		No	Unsure	Yes
13	I planned to get pregnant at this time	1	2	3
14.	It just happened	1	2	3
15.	I meant to avoid pregnancy at this time	1	2	3
16.	I wanted to get pregnant at this time	1	2	3
17.	My method of family planning failed	1	2	3

18. Which method of family planning did you last use before you became pregnant?

	Please Circle
Natural/Rhythm Method/Safe Period	1
Pill	2
Condom	3
Foam, chemical barrier agents	4
Withdrawal	5
Diaphragm	6
I.U.D. (intrauterine device)	7
Other, (e.g. Injection) Specify ______________________	8
No method of family planning used	9

	No	Yes
19. Did you have any problems becoming pregnant?	1	2

20. How long had you been trying to become pregnant? .... Years .... Months
(If you were not trying to become pregnant please put "0".)

Now we are interested in how you have been feeling recently .

PLEASE CIRCLE ONE NUMBER FOR EACH STATEMENT FOR QUESTIONS 21 - 27.

	All the Time	Most of the Time	Some of the Time	Rarely	Never
21. I have worried about every little thing	1	2	3	4	5
22. I have been so miserable that I have had difficulty sleeping	1	2	3	4	5
23. I have been breathless or had a pounding of my heart	1	2	3	4	5
24. I have been so worked up that I couldn't sit still	1	2	3	4	5
25. I have been depressed without knowing why	1	2	3	4	5
26. I have gone to bed not caring if I never woke up	1	2	3	4	5
27. For no good reason I have had feelings of panic	1	2	3	4	5

PLEASE CIRCLE ONE NUMBER FOR EACH STATEMENT FOR QUESTIONS 28 - 38.

		All the Time	Most of the Time	Some of the Time	Rarely	Never
28.	I have been so low in spirit that I have sat up for ages doing absolutely nothing	1	2	3	4	5
29.	I have had a pain or tense feeling in my neck or head	1	2	3	4	5
30.	The future seems hopeless	1	2	3	4	5
31.	Worrying has kept me awake at night	1	2	3	4	5
32.	I have lost interest in just about everything	1	2	3	4	5
33.	I have been so anxious that I couldn't make up my mind about the simplest thing	1	2	3	4	5
34.	I have been so depressed that I have thought of doing away with myself	1	2	3	4	5
35.	In general, I am usually tense or nervous	1	2	3	4	5
36.	There is a great amount of nervous strain connected with my daily activities. I am always under pressure	1	2	3	4	5
37.	At the end of the day I am completely exhausted mentally and physically	1	2	3	4	5
38.	My daily activities are extremely trying and stressful	1	2	3	4	5

39. How satisfied are you with your life as a whole these days? Would you say you are:

PLEASE CIRCLE ONE NUMBER

Very satisfied	1
Satisfied	2
Dissatisfied	3
Very dissatisfied	4

40. How would you say you feel these days? Would you say you are:

PLEASE CIRCLE ONE NUMBER

Very happy	1
Fairly happy	2
Not too happy	3

Sometimes events happen which can affect the way you live. We would like to know if any of the following have happened to you IN THE LAST 6 MONTHS.

PLEASE CIRCLE ONE NUMBER FOR EACH QUESTION FROM 41 - 49.

	No	Yes
41. Has someone close to you died or been seriously ill?	1	2
42. Have you had a big problem with your own health?	1	2
43. Have you had serious disagreements with your partner?	1	2
44. Have you had serious disagreements with someone else close to you?	1	2
45. Have you had serious financial problems?	1	2
46. Has your partner had a major change in his job situation?	1	2
47. Have you had a major change in your job situation?	1	2
48. Have you had any serious problems with housing or accommodation?	1	2
49. Have you or your partner had a problem with the law ?	1	2

At times there are arguments and upsets in every relationship.

How well do the following statements describe the relationship between you and your partner.

PLEASE CIRCLE ONE NUMBER FOR EACH STATEMENT FOR QUESTIONS 50 - 56.

	No Partner	All the Time	Most of the Time	Some of the Time	Rarely	Never
50. In general, would you say that things between you and your partner are going well?	1	2	3	4	5	6
51. How often do you think about divorce, separation or terminating your relationship?	1	2	3	4	5	6
52. How often do you or your partner leave the house after a fight?	1	2	3	4	5	6
53. Do you find it easy to confide in your partner?	1	2	3	4	5	6
54. Do you ever regret that you married (or lived together) ?	1	2	3	4	5	6
55. How often do you and your partner quarrel?	1	2	3	4	5	6
56. How often do you and your partner "get on each other's nerves"?	1	2	3	4	5	6

57. How satisfied are you with your relationship with your husband/partner. Would you say you are:

	PLEASE CIRCLE ONE NUMBER
Very satisfied	1
Satisfied	2
Dissatisfied	3
Very dissatisfied	4
No husband/partner	5

Here is a set of questions about how you lived BEFORE YOU BECAME PREGNANT.

PLEASE CIRCLE ONE NUMBER

58. How many hours sleep did you usually get a night?

9 hours or more	1
8 hours	2
7 hours	3
6 hours or less	4

59. How often did you eat breakfast?

Almost every day	1
Sometimes	2
Rarely or never	3

60. How often did you eat in between your regular meals?

Almost every day	1
Sometimes	2
Rarely or never	3

Here is a list of things that people do in their spare time.
BEFORE YOU BECAME PREGNANT how often did you do any of these things?

PLEASE CIRCLE ONE NUMBER FOR EACH QUESTION FROM 61 - 65.

	Often	Sometimes	Never
61. Active sports	1	2	3
62. Swimming or taking long walks	1	2	3
63. Taking week-end trips in the car	1	2	3
64. Working in the garden	1	2	3
65. Doing physical exercises	1	2	3

66. On average about how many hours a day did you watch T.V.?

PLEASE CIRCLE ONE NUMBER

Watch more than 7 hours	1
Watch about 5 - 7 hours	2
Watch about 3 - 5 hours	3
Watch about 1 - 3 hours	4
Watch less than 1 hour	5
Never watch T.V. at all	6

BEFORE YOU BECAME PREGNANT

PLEASE CIRCLE ONE NUMBER FOR EACH QUESTION FROM 67 - 71.

67. How many times a week did you smoke cigarettes before you became pregnant?

Every day	1
Every few days	2
Once or so only	3
Did not smoke at all	4

68. How many cigarettes did you usually smoke per day before you became pregnant?

50 or more per day	1
30 - 49 per day	2
20 - 29 per day	3
10 - 19 per day	4
1 - 9 per day	5
Nil smoked	6

69. How often did you usually drink alcohol before you became pregnant?

Daily	1
A few times a week	2
A few times a month	3
A few times a year	4
Rarely	5
Never	6

70. How much alcohol did you usually drink at those times?

Seven or more glasses	1
Five or six glasses	2
Three or four glasses	3
One or two glasses	4
Never drank	5

71. Have you every tried cannabis, marihuana, pot, etc. before you became pregnant?

No	1
Yes	2

Many women smoke and drink during their pregnancies. We would like to know more about women coming to our hospital.

PLEASE CIRCLE ONE NUMBER FOR EACH QUESTION FROM 72 – 76

72. In the last week how often did you smoke cigarettes?

Every day	1
Every few days	2
Once or so only	3
Did not smoke at all	4

73. In the last week how many cigarettes did you usually smoke per day?

50 or more per day	1
30 - 49 per day	2
20 - 29 per day	3
10 - 19 per day	4
1 - 9 per day	5
Nil smoked	6

74. How often do you drink alcohol since becoming pregnant?

Daily	1
A few times a week	2
A few times a month	3
A few times a year	4
Rarely	5
Never	6

75. How much alcohol do you usually drink at those times?

Seven or more glasses	1
Five or six glasses	2
Three or four glasses	3
One or two glasses	4
Less than one glass	5
Never drink	6

76. When you drink alcohol what part of the time do you have at least 5 glasses?

Nearly every time	1
More than half the time	2
About half the time	3
Less than half the time	4
Once in a while	5
Never	6

PLEASE CIRCLE ONE NUMBER FOR EACH QUESTION.

77. Since becoming pregnant how many cups of coffee do you drink per day, on average?

7 cups or more	1
4 - 6 cups per day	2
2 - 3 cups per day	3
1 cup per day	4
None	5

78. Since becoming pregnant how many cups of tea do you drink per day, on average?

7 cups or more	1
4 - 6 cups per day	2
2 - 3 cups per day	3
1 cup per day	4
None	5

79. How often did you use cannabis, marihuana, pot etc. in the last month?

Every day	1
Every few days	2
Once or so	3
Not in the last month	4
Never	5

Now here are some questions about your background.

PLEASE CIRCLE ONE NUMBER FOR EACH QUESTION FROM 80 - 83.

80. At what level did you complete your education?

Opportunity School	1
Primary School	2
Started Secondary School	3
Completed Grade 10 - (age 15 - 16 years)	4
Completed Grade 12 - (age 17 - 18 years)	5
College (e.g. Business, Nursing, Secretarial, Teachers')	6
University	7
Other (please specify)	8

81. At what level did you partner complete his education?

Not applicable (no partner)	1
Opportunity School	2
Primary School	3
Started Secondary School	4
Completed Grade 10 - (age 15 - 16 years)	5
Completed Grade 12 - (age 17 - 18 years)	6
College (e.g. Business, Trade, Secretarial, Teachers')	7
University	8
Other (please specify)	9

82. Are you presently:

Fully employed	1
Self-employed	2
Employed part-time	3
Unemployed	4
On Pension, e.g. Invalid, Widow’s, Supporting Mother’s	5
Student	6
Housewife	7
Other (please specify)	8

83. Is your partner presently:

Not applicable (no partner)	1
Fully employed	2
Self-employed	3
Employed part-time	4
Unemployed	5
On Invalid Pension	6
Student	7
Other (please specify)	8

84. What work do you usually do for a living? (Please state in detail, for example if clerk - what type of clerk?)

____
____

85. What work does your partner usually do for a living? Please state in detail. If no partner, write '0'.

____
____

86. Does his job involve his sleeping away from home?

Not applicable (no partner)	1
Several nights a week	2
One night a week	3
About once a month	4
Rarely	5
Never	6

87. What is, or was, your father's main occupation? Please answer in full detail.

____
____

88. What is, or was, your mother's main occupation? Please answer in full detail.

____
____

PLEASE CIRCLE ONE NUMBER FOR <u>EACH</u> QUESTION.

89. What is your present marital status?

Single	1
Living together/De facto	2
Married	3
Separated/divorced	4
Widowed	5
Other (please specify)	6

____

90. On the list below could you circle the number closest to YOUR WHOLE FAMILY'S INCOME (gross) including spouse's income, child endowment, etc. If unsure circle the number closest to the amount you think may be correct.

Per Year	Per Week	
$0 - $2,599	$0 - $49	1
$2,600 - $5,199	$50 - $99	2
$5,200 - $10,399	$100 - $199	3
$10,400 - $15,599	$200 - $299	4
$15,600 - $20,799	$300 - $399	5
$20,800 - $25,999	$400 - $499	6
$26,000 or more	$500 or more	7

PLEASE CIRCLE ONE NUMBER FOR EACH QUESTION FROM 91 - 96.

91. In what religion were you brought up?

Catholic	01
Church of England	02
No religion/Agnostic/Atheist	03
Other (please specify)	04

____

92. What is your present religion?

Catholic	01
Church of England	02
No religion/Agnostic/Atheist	03
Other (please specify)	04

____

93. How often do you go to church?

Weekly	1
Monthly	2
Less than once a month	3
Never	4

94. Do you believe in God?

No	1
Yes	2
Do not know	3

95. Do you live in a:

House (detached/semi/terrace)	1
Flat/Unit	2
Share part of a house or flat	3
Caravan	4
Other	5

96. Do you:

Own your own accommodation outright	1
Live in own accommodation with loan or mortgage	2
Occupy accommodation in connection with your own or spouse's occupation	3
Rent your own accommodation	4
Live with parents or parents-in-law	5
Live with other family members (e.g. brother, sister)	6
Live in a hostel or refuge	7
Other (please specify)	8

____

97. Where you live how many rooms are available for your use? ………………… rooms
(includes bathroom, toilet, kitchen and all enclosed areas.)

98. In which country were you born? …………

99. How long have you been in Australia? Years ... Months

	No	Yes
100. Have you any trouble understanding English?	1	2

	No	Yes
101. Have you had a previous marriage?	1	2

102. Were you personally receiving any of the following benefits at any time in the six months before you became pregnant?

PLEASE CIRCLE ONE NUMBER

No benefit received	1
Unemployment benefit	2
Sickness benefit	3
Supporting Parents benefit	4
Invalid Pension	5
Widow's Pension	6

103. To which of the following groups do you belong?

PLEASE CIRCLE THE MOST APPROPRIATE NUMBER.

Australian Aborigine	1
Maori/Islander	2
Asian	3
White	4
Other (please specify)	5

____

The following questions are about your partner. If you have no partner put '0'.

104. In which country was your partner born? ....

105. How long has your partner been in Australia? ....Years ... .Months

106. How long has your present marriage or relationship existed? ....

	No	Yes	No Partner
107. Has your partner had a previous marriage?	1	2	3

108. What is your husband or partner's age? ....
If no partner put '0'.

109. To which of the following groups does your partner belong?

PLEASE CIRCLE THE MOST APPROPRIATE NUMBER.

Australian Aborigine 1
Maori/Islander.....................................2
Asian 3
White 4
Other (please specify) 5

____

	No	Yes
110. Do you live alone?	1	2
111. Do you live with your partner? (If no partner please circle "l".)	1	2
112. Do you live with your children? (if no children please circle "1".)	1	2

113. Did you fill this questionnaire in:

Alone	1
With the help of a friend	2
With the help of your partner	3
With the help of someone at the clinic	4
