# Supplementary material for: Effect of maternal smoking in pregnancy and childhood on child and adolescent sleep outcomes to 21 years: a birth cohort study
Source: BMC Pediatr. 2019 Mar 6;19:70. doi: 10.1186/s12887-019-1439-1 (PMC6402153; doi:10.1186/s12887-019-1439-1)
Supplement: Supplementary file 2 — MUSP obstetric. (PDF 313 kb) [file 12887_2019_1439_MOESM2_ESM.pdf]

MUSP OBSTETRIC DATA SHEET - A

PHASE: 5

NAME: ______________________________ CODE NO: _______________

U.R. NO:

PAST OBSTETRIC HISTORY:

1. (AN) Total No. of previous pregnancies

	Yr	Gest'n	Bth Wt (gms)	Sex	Delivery	Outcome
2.						
3.						
4.						
5.						
6.						
7.						
8.						
9.						
10.						
11.						

CODE

SEX	DELIVERY	OUTCOME
1. Male	1. Normal	1. Live (at present)
2. Female	2. Forceps	2. Miscarriage
3. Male - Twin	3. Breech	3. T.O.P.
4. Female - Twin	4. Caesarean section	4. Ectopic
		5. S.B.
		6. N.N.D.
		7. Death (l mth-1yr)
		8. Death(1 yr))
		9. Adoption,Fostered

Present Pregnancy

12	(AN)	Height (to nearest cms)	
13		Height - patients estimate (cms)	
14	(AN)	Pre-pregnant weight (to nearest tags)	
15		Pre-pregnant weight - patients estimate (kgs)	
16		Height of baby's father (cms)	
17		Weight of baby's father (kgs)	
18	(AN)	Date of 1st Antenatal visit	
19		Gestation at 1st Antenatal visit	
20	(AN)	Weight at 1st visit (to nearest kg)	
21	(AN)	Date of maximum recorded weight	
22		Gestation at maximum recorded weight	
23	(AN)	Maximum recorded weight (to nearest kg)	
24	(AN)	Date of last Antenatal visit	
25		Gestation at last Antenatal visit	
26	(AN)	Weight at last Antenatal visit (to nearest kg)	
27	(AN)	Lowest recorded haemoglobin (gms)	

Delivery

28 (HS) Hour of Delivery (to next o'clock)

12.01 a.m. -- 1.00 a.m. = 01
1.01 a.m. - 2.00 a.m. = 02
11.01 p.m. -12.00 midnight = 24

29 (HS) Duration of Labour

1st Stage (to nearest hour)

2nd Stage (in minutes)

30 (AD2) Insurance

31 (AD5) Date of birth of mother (month and year)

32 (AD6) Suburb/Local Authority Code

33 (AD7) H.M.O.

34 (AD47) Placental Weight (gms)

35 (AD51) Method of Induction

36 (AD52) Augmentation of Labour

37 (AD54) Number of Infants Delivered

38 (AD56) Anaesthesia (N_20 excluded)

39 (AD57) Fetal Distress

40 (AD60) Intravenous Infusion

41 (AD70) Date of Discharge after Delivery

42 (AD71) Number of Antenatal Admissions

43	(ND1)	Date of Delivery
44	(ND2)	Birth Weight (gms)
45	(ND3)	Sex
46	(ND4)	Baby in Multiple Pregnancy
47	(ND5)	Presentation at Delivery
48	(ND6)	Method of. Delivery
49	(ND7)	Apgar at 1 minute
50	(ND8)	Apgar at 5 minutes
51	(ND9)	Time to establish respiration
52	(ND10a)	Resuscitation given – mechanical
53	(ND10b)	Resuscitation given – drug
54	(ND11)	Length of baby (crown - heel)
55	(ND15)	Head Circumference (cms)
56	(ND16)	Jaundice
57	(ND17)	Highest Bilirubin
58	(ND20)	Congenital Malformation
59	(ND24)	Date of Discharge or Death of Baby
60	(ND26)	Infants Survival in Hospital
61	(ND27)	Disposal

OBSTETRIC DATA SHEET – B

PHASE: 5

NAME: ________________________________ CODE NO: __________________

U.R. NO:

1 Coded by

2 Gestation at Delivery

3 Dates - Not certain ....……………..….. 1
Certain ....………………..….. 2

4 Date of 1st Contact with Mater Mothers' Hospital

5. Booking - Apparent Date of 1s t Visit Anywhere
Gestation - 20 weeks or less 1
Gestation - 21 - 30 weeks 2
Gestation - 31 weeks or more 3
Unbooked 4
Not known 9

6. Attendance - Antenatal Care Anywhere
Regular 1
Irregular (< 50% of scheduled A/N visits) 2
Nil 3
Not applicable 4
Not known 9

7. Information re Booking/Attendance
Not certain 1
Certain 2

8. Antenatal Admissions

No. of antenatal admissions - < 3 days

3 - 7 days

> 7 days

9. Complications this pregnancy — Gestation (1st diagnosed) — Severity

	Gestation (1st diagnosed)	Severity
(a) Essential Hypertension	☐☐	☐
(b) Pre-eclampsia	☐☐	☐
(c) Bleeding - abruptio placenta	☐☐	☐
- antepartum haemorrhage	☐☐	☐
- placenta praevia	☐☐	☐
- unclassified	☐☐	☐

10. Diseases significant to pregnancy — Disease — Severity

	Disease	Severity
(a) Pre-existing – specify ______________ ________________________________	☐☐	☐
(b) Newly developed – specify _________ ______________________________	☐☐	☐

11 Number of admissions to Labour Ward at <37 completed weeks of pregnancy for premature labour ☐

12. Analgesics — No. of Doses

	No. of Doses
Haloperidol, droperidol	☐
Morphine	☐
Omnopon	☐
Pethidine	☐
Stemetil	☐
Valium	☐
Others e.g. largactil, sparine etc.	☐

13. Fetal distress

	No	Yes	
As indication for operative delivery	1	2	☐
Meconium	1	2	☐
Fetal Heart Monitoring	1	2	☐

14. Syntocinon

Total time (to nearest hour)		☐☐
Highest concentration	- units	☐☐
	- drops	☐☐

15. Outcome at Delivery

Alive.. 1
Dead (antepartum macerated) 2
(antepartun fresh) 3
(antepartum unknown) 4
(intrapartum) 5
(immediate postpartum
i.e. < 1hour) 6

☐

16. No. of days in Intensive Care Nursery ☐☐

17. Significant Neonatal Problems

		No	Yes	
(a)	Observation only (no other reason)	1	2	☐
(b)	Prematurity	1	2	☐
(c)	Small for gestational age	1	2	☐
(d)	Respiratory problems	1	2	☐
(e)	Central Nervous System Problems	1	2	☐
(f)	Blood Coagulation problems	1	2	☐
(g)	Feeding problems	1	2	☐
(h)	Trauma	1	2	☐
(i)	Jaundice	1	2	☐
(j)	Metabolic problems	1	2	☐
(k)	Congenital Abnormality	1	2	☐
(1)	Infection	1	2	☐
(m)	Cardiac	1	2	☐
(n)	Others - please specify ____________________	1	2	☐

____

18. Outcome

Alive 1
Alive with obvious residual handicap 2
Dead 3

☐

19. Age at Death

No. of days (<24 hours = 01) ☐☐
