# Supplementary material for: Effect of maternal smoking in pregnancy and childhood on child and adolescent sleep outcomes to 21 years: a birth cohort study
Source: BMC Pediatr. 2019 Mar 6;19:70. doi: 10.1186/s12887-019-1439-1 (PMC6402153; doi:10.1186/s12887-019-1439-1)
Supplement: Supplementary file 3 — MUSP maternal 6 months. (PDF 326 kb) [file 12887_2019_1439_MOESM3_ESM.pdf]

MUSP Maternal Questions 6 Months

PHASE: 3

CODE NO: __________________

DATE RETURNED: __________________

FIRST, WE WOULD LIKE TO ASK SOME QUESTIONS ABOUT YOUR BABY.

How often does your baby have the following problems?

PLEASE CIRCLE ONE NUMBER FOR EACH STATEMENT FROM 1 - 9.

		Almost every day	A few times a week	A few times a month	Rarely	Never
1.	Colic	1	2	3	4	5
2.	Sleeplessness	1	2	3	4	5
3.	Vomiting	1	2	3	4	5
4.	Diarrhoea or constipation	1	2	3	4	5
5.	Feeding problem	1	2	3	4	5
6.	Skin problems e.g. nappy rash, eczema	1	2	3	4	5
7.	Overactivity	1	2	3	4	5
8.	Cough, cold or runny nose	1	2	3	4	5
9.	Convulsions or fits	1	2	3	4	5

10. Since the birth how many times have you taken your baby to a Maternal Health and Child Welfare Clinic?

(Please write "0" if never)

………………..times

11. Since the birth how many times have you sought medical attention for your baby?
(Please write "0" if never)

…………………..times

12. Since the birth how many times has your baby had to be admitted into hospital?

(Please write "0" if never)

……………………..times

13. (a) Has your baby had any serious illness or handicap since first leaving hospital?

	Please circle one number
No	1
Yes	2

(b) If YES, what is it? (Please describe)

____

____

14. (a) Does your baby NOW have any serious illness or handicap?

	Please circle one number
No	1
Yes	2

(b) If YES, what is it? (Please describe)

____

____

15. What is your baby's weight now?

____

16. In the first six months did you mostly feed on demand or at regular intervals?

	Please circle one number
On demand	1
At regular intervals	2

17. How long did you breast feed?

	Please circle one number
Still breast feeding	1
4 months to 6 months	2
7 weeks to 3 months	3
3 weeks to 6 weeks	4
2 weeks or less	5
Not at all	6

Now here are some questions about <u>what your baby does.</u> Your child is not expected to be able to do everything the questions ask.

PLEASE CIRCLE ONE ANSWER FOR <u>EACH</u> QUESTION FROM 18 - 23.

		NO	YES
18.	When your baby is lying on his/her back, does he/she move each arm or leg as easily as the other?	1	2
19.	When your baby is lying on his/her back, does he/she look at you and watch your face?	1	2
20.	Does your baby make sounds such as gurgling, cooing, babbling or other noises except crying?	1	2
21.	When your baby is on his/her back, does he/she follow your movement by turning his/her head?	1	2
22.	When you smile and talk to your baby does he/she smile back at you?	1	2
23.	When your baby is on his/her stomach on a flat surface can he/she lift his/her head off the bed or surface?	1	2

24. How do you feel about your baby's development (e.g. rolling over, sitting up, etc.)?

	<u>Please circle one number</u>
Very satisfied	1
Somewhat satisfied	2
Neither satisfied nor worried	3
Somewhat worried	4
Very worried	5

25. How many hours per week does someone else look after the baby for you?

	<u>Please circle one number</u>
More than 20 hours a week	1
11 to 20 hours a week	2
5 to 11 hours a week	3
Less than 4 hours a week	4
Never	5

26. How many times has your baby had a triple antigen immunisation (i.e. Diphtheria, Whooping Cough and Tetanus)?

	<u>Please circle one number</u>
Four times	1
Three times	2
Twice	3
Once	4
Nil yet	5

THE FOLLOWING ARE SOME QUESTIONS ASKING HOW YOU FEEL ABOUT YOUR BABY.
How well do the following statements describe how you feel?

PLEASE CIRCLE ONE NUMBER FOR EACH STATEMENT FROM 27 - 38.

		Strongly Agree	Agree	Neutral	Disagree	Strongly Disagree
27.	Caring for my baby is very satisfying.	1	2	3	4	5
28.	I always pick my baby up as soon as he/she starts to cry.	1	2	3	4	5
29.	I don't think it is worth talking to my baby until he/she gets older.	1	2	3	4	5
30.	I want to see more of my baby.	1	2	3	4	5
31.	I feel so angry that sometimes I could smack my baby.	1	2	3	4	5
32.	I try to encourage my baby to be interested in what is going on around him/her.	1	2	3	4	5
33.	I think my baby should get his/her own way.	1	2	3	4	5
34.	My baby makes me too tired.	1	2	3	4	5
35.	My baby likes me talking to him/her.	1	2	3	4	5
36.	I try to put my baby to bed at the same time each day.	1	2	3	4	5
37.	My baby is so good I hardly know he/she is there.	1	2	3	4	5
38.	I am too busy to spend a lot of time playing with my baby.	1	2	3	4	5

PLEASE CIRCLE ONE NUMBER FOR EACH STATEMENT FROM 39 - 44.

	Strongly Agree	Agree	Neutral	Disagree	Strongly Disagree
39. I don't allow my baby to rule my life.	1	2	3	4	5
40. I sometimes feel like hitting my baby.	1	2	3	4	5
41. I spend a lot of time teaching my baby to recognise things.	1	2	3	4	5
42. I am going to try and toilet train my baby before he/she is one year old.	1	2	3	4	5
43. I love to play with my baby.	1	2	3	4	5
44. I feel fed up looking after my baby all day.	1	2	3	4	5

45. Have you had your baby circumcised?

	Please circle one number
Not applicable (my baby is a girl)	1
No	2
Yes	3

46. A LOT HAS BEEN WRITTEN ABOUT "POSTPARTUM BLUES" AND DEPRESSION BUT WE STILL DON'T KNOW THE TRUE SITUATION.

Which of the following best describes how you felt?

	Please circle one number
I had no feelings of blues or depression at all	1
I felt slightly depressed	2
I was moderately depressed	3
I was very depressed	4

47. For how long did you feel blue or depressed?

	Please circle one number
Not at all	1
A few days	2
A few months	3
I am still depressed	4

Now we are interested in <u>how you have been feeling recently.</u>

PLEASE CIRCLE ONE NUMBER FOR <u>EACH</u> STATEMENT FROM 48 - 60.

		All the Time	Most of the Time	Some of the Time	Rarely	Never
48.	I have worried about every little thing.	1	2	3	4	5
49.	I have been so miserable that I have had difficulty sleeping.	1	2	3	4	5
50.	I have been breathless or had a pounding of my heart.	1	2	3	4	5
51.	I have been so worked up that I couldn't sit still.	1	2	3	4	5
52.	I have been depressed without knowing why.	1	2	3	4	5
53.	I have gone to bed not caring if I never woke up.	1	2	3	4	5
54.	For no good reason I have had feelings of panic.	1	2	3	4	5
55.	I have been so low in spirit that I have sat up for ages doing absolutely nothing.	1	2	3	4	5
56.	I have had a pain or a tense feeling in my neck or head.	1	2	3	4	5
57.	The future seems hopeless.	1	2	3	4	5
58.	Worrying has kept me awake at nights.	1	2	3	4	5
59.	I have lost interest in just about everything.	1	2	3	4	5
60.	I have been so anxious that I couldn't make up my mind about the simplest thing.	1	2	3	4	5

PLEASE CIRCLE ONE NUMBER FOR EACH STATEMENT FROM 61 - 65.

	All the Time	Most of the Time	Some of the Time	Rarely	Never
61. I have been so depressed that I have thought of doing away with myself.	1	2	3	4	5
62. In general, I am usually tense or nervous.	1	2	3	4	5
63. There is a great amount of nervous strain connected with my daily activities. I am always under pressure.	1	2	3	4	5
64. At the end of the day I am completely exhausted, mentally and physically.	1	2	3	4	5
65. My daily activities are extremely trying and stressful.	1	2	3	4	5

66. How satisfied are you with your life as a whole these days?

	Please circle one number
Very satisfied	1
Satisfied	2
Dissatisfied	3
Very dissatisfied	4

67. How would you say you feel these days? Would you say you are:

	Please circle one number
Very happy	1
Fairly happy	2
Not too happy	3

68. IN AN EARLIER QUESTIONNAIRE WE ASKED YOU SOME QUESTIONS ABOUT HOW MANY FRIENDS AND RELATIVES YOU COULD COUNT ON.

Has the number of people you can turn to for help or support changed in the last six months?

	Please circle one number
There are less people	1
There are the same number of people	2
There are more people	3

69. Since the birth how many times have you sought medical attention for yourself?

(Please write "0" if never)

.... times

70. In the <u>last week</u> have you used any medication to relieve pain?

Please circle one number

No	1
Yes	2

71. In the <u>last week</u> how often did you use these pain relievers?

Please circle one number

Every day	1
Every few days	2
Once or so	3
Not at all	4

72. In the <u>last week</u> have you used any sedatives, sleeping pills, tranquillisers, anti-depressants or other drugs to help you relax?

Please circle one number

No	1
Yes	2

73. In the <u>last week</u> how often did you use these?

Please circle one number

Every day	1
Every few days	2
Once or so	3
Not at all	4

74. In the <u>last week</u> how often did you smoke cigarettes?

	Please circle one number
Every day	1
Every few days	2
Once or so only	3
Did not smoke at all	4

75. In the <u>last week</u> how many cigarettes did you usually smoke per day?

	Please circle one number
50 or more per day	1
30 - 49 per day	2
20 - 29 per day	3
10 - 19 per day	4
1 - 9 per day	5
Nil smoked	6

76. How often have you drunk alcohol since you had the baby?

	Please circle one number
Daily	1
A few times a week	2
A few times a month	3
A few times a year	4
Rarely	5
Never	6

77. How much alcohol do you usually drink at those times?

	Please circle one number
Seven or more glasses	1
Five or six glasses	2
Three or four glasses	3
One or two glasses	4
Less than one glass	5
Never drink	6

78. In the <u>last month</u> how often did you use cannabis, marihuana, pot etc.?

	Please circle one number
Every day	1
Every few days	2
Once or so	3
Not in the last month	4
Never	5

At times there are arguments and upsets in every relationship.

How well do the following statements describe the relationship between you and your partner.

PLEASE CIRCLE ONE NUMBER FOR EACH STATEMENT FOR QUESTIONS 79 - 85.

	No Partner	All the time	Most of the time	Some of the time	Rarely	Never
79. In general, would you say that things between you and your partner are going well?	1	2	3	4	5	6
80. How often do you think about divorce, separation or terminating your relationship?	1	2	3	4	5	6
81. How often do you or your partner leave the house after a fight?	1	2	3	4	5	6
82. Do you find it easy to confide in your partner?	1	2	3	4	5	6
83. Do you ever regret that you married (or live together) ?	1	2	3	4	5	6
84. How often do you and your partner quarrel?	1	2	3	4	5	6
85. How often do you and your partner get on each other's nerves?	1	2	3	4	5	6

86. How satisfied are you with your relationship with your husband/partner? Would you say you are:

	Please circle one number
Very satisfied	1
Satisfied	2
Dissatisfied	3
Very dissatisfied	4
No husband/partner	5

87. What is your present marital status?

	Please circle one number
Single	1
Living together/De facto	2
Married	3
Separated/divorced	4
Widowed	5
Other (please specify) ______________________	6

88. Do you plan to become pregnant in the next six months?

	Please circle one number
No	1
Yes	2
I think I am pregnant	3

89. Which of the following are you using as your main method of family planning?

	Please circle one number
Natural/Rhythm method/Safe Period	1
Pill	2
Condom	3
Foam, chemical barrier agents	4
Withdrawal	5
Diaphragm	6
I.U.D. (intrauterine device)	7
Tubal ligation (female sterilization	8
Vasectomy (male sterilization)	9
None	10
Other (e.g. injection)	11
Please specify ______________________	

90. How many children are there in your household altogether?

	Please circle one number
One	1
Two	2
Three	3
Four	4
Five	5
Six	6
Seven	7
Eight	8
Nine or more	9

91. How many more children would you like to have?

	Please circle one number
None	0
One	1
Two	2
Three	3
Four	4
Five	5
Six	6
Seven	7
Eight or more	8

92. Are you presently:

Please circle one number

Fully employed	1
Self-employed	2
Employed part-time	3
Unemployed	4
On pension e.g. invalid, widows', supporting mothers'	5
Student	6
Housewife	7
Other (please specify)	8

____

93. Is your partner presently:

Please circle one number

Not applicable (no partner)	1
Fully employed	2
Self-employed	3
Employed part-time	4
Unemployed	5
On Invalid Pension	6
Student	7
Other (please specify)	8

____

94. Do you plan to get a job in the next 6 months?

Please circle one number

No	1
I am already working	2
Yes - full time	3
Yes - part time	4

95. On the list below could you circle the number closest to your whole family's income (gross) including spouse's income, child endowment, etc.if unsure, circle the number closest to the amount you think may be correct.

Per Year	Per Week	
$ 0 - $ 2,599	.......$ 0 - $ 49	1
$ 2,600 - $ 5,199	......$ 50 - $ 99	2
$ 5,200 - $10,399	......$100 - $199	3
$10,400 - $15,599	......$200 - $299	4
$15,600 - $20,799	......$300 - $399	5
$20,800 - $25,999	......$400 - $499	6
$26,000 or more	......$500 or more	7

96. Have you personally been receiving any of the following benefits in the last six months?

	Please circle one number
No benefit received	1
Unemployment benefit	2
Sickness benefit	3
Supporting Parent's benefit	4
Invalid Pension	5
Special Pregnancy benefit	6
Widow's Pension	7
Other (please specify)	8

____

97. Do you still have the same partner you had when the baby was born?

	Please circle one number
No partner	1
No	2
Yes	3

98. How many times have you changed your address since the birth of your baby?
(If you have not changed your address please put "0"

...................................times

99. Do you live alone?

No	1
Yes	2

100. Do you live with your partner?

No	1
Yes	2

101. Do you live with your child or children?

No	1
Yes	2

102. Do you live with your parents?

No	1
Yes	2

103. Did you fill this questionnaire in:

Alone	1
With the help of a friend	2
With the help of your partner	3
With the help of someone from the clinic	4
