# Supplementary material for: Effect of maternal smoking in pregnancy and childhood on child and adolescent sleep outcomes to 21 years: a birth cohort study
Source: BMC Pediatr. 2019 Mar 6;19:70. doi: 10.1186/s12887-019-1439-1 (PMC6402153; doi:10.1186/s12887-019-1439-1)
Supplement: Supplementary file 4 — MUSP maternal 5 years. (PDF 294 kb) [file 12887_2019_1439_MOESM4_ESM.pdf]

PHASE: | 5 | 2 |

CODE NO: ______________

DATE: ______________

1. In general, over the last year, do you think your health has been?
(please circle one number)

Excellent	1
Good	2
Fair	3
Poor	4

2. How often have you been to a doctor about your own health in the last year?
(please circle one number)

Never	1
Once	2
Twice	3
Three times	4
Four times	5
Five times or more	6

3. What health problems have you had in the last year?
(Write "0" if none).

____ [|]

____

____ [|]

4. Does your health limit the kind or amount of vigorous activities you can do?
(please circle one number)

No	1
Yes, sometimes	2
Yes, often	3

5. Please list the age and sex of all children in the household:

Child	Age	Sex
1		
2		
3		
4		
5		
6		
7		
8		
9		

10		

NOW WE ARE INTERESTED IN HOW YOU HAVE BEEN FEELING RECENTLY.

PLEASE CIRCLE ONE NUMBER FOR EACH STATEMENT.

		All the time	Most of the time	Some of the time	Rarely	Never
6.	I have worried about every little thing.	1	2	3	4	5
7.	I have been so miserable that I have had difficulty sleeping.	1	2	3	4	5
8.	I have been breathless or had a pounding of my heart.	1	2	3	4	5
9.	I have been so worked up that I couldn't sit still.	1	2	3	4	5
10.	I have been depressed with-out knowing why.	1	2	3	4	5
11.	I have gone to bed not caring if I never woke up.	1	2	3	4	5
12.	For no good reason I have had feelings of panic.	1	2	3	4	5
13.	I have been so low in spirit that I have sat up for ages doing absolutely nothing.	1	2	3	4	5
14.	I have had a pain or tense feeling in my neck or head.	1	2	3	4	5
15.	The future seems hopeless.	1	2	3	4	5
16.	Worrying has kept me awake at night.	1	2	3	4	5

		All the time	Most of the time	Some of the time	Rarely	Never
17.	I have lost interest in just about everything.	1	2	3	4	5
18.	I have been so anxious that I couldn't make up my mind about the simplest thing.	1	2	3	4	5
19.	I have been so depressed that I have thought of doing away with myself.	1	2	3	4	5
20.	In general, I am usually tense or nervous.	1	2	3	4	5
21.	There is a great amount of nervous strain connected with my daily activities. I am always under pressure.	1	2	3	4	5
22.	At the end of the day I am completely exhausted mentally and physically.	1	2	3	4	5
23.	My daily activities are extremely trying and stressful.	1	2	3	4	5

24. How satisfied are you with your life as a whole these days? Would you say you are: (please circle one number)

Very satisfied	1
Satisfied	2
Dissatisfied	3
Very dissatisfied	4

25. How would you say you feel these days? Would you say you are: (please circle one number)

Very happy	1
Fairly happy	2
Not too happy	3
Very unhappy	4

WE ARE INTERESTED IN ANY CHANGES IN YOUR LIFE SINCE YOUR 5-YEAR-OLD CHILD WAS BORN.
HAVE ANY OF THE FOLLOWING HAPPENED TO YOU?

CIRCLE A NUMBER EACH TIME THIS HAS HAPPENED.

		No	Yes - it happened				
			1 yr. ago	2 yrs. ago	3 yrs. ago	4 yrs. ago	5 yrs. ago
26.	Have you had a major change in your job situation?	0	1	2	3	4	5
27.	Has your partner had a major change in his job situation?	0	1	2	3	4	5
28.	Have you had trouble at work?	0	1	2	3	4	5
29.	Has your partner had trouble at work?	0	1	2	3	4	5
30.	Have you had to cut back on food because you couldn't afford the cost?	0	1	2	3	4	5
31.	Have you or your partner had a problem with the police?	0	1	2	3	4	5
32.	Has your partner died?	0	1	2	3	4	5
33.	Has a close friend died?	0	1	2	3	4	5
34.	Have you been divorced?	0	1	2	3	4	5

			Yes - it happened				
		No	1 yr. ago	2 yrs. ago	3 yrs. ago	4 yrs. ago	5 yrs. ago
35.	Have you and your partner separated?	0	1	2	3	4	5
36.	Have you changed partners?	0	1	2	3	4	5
37.	Have you had any serious dis-agreements with your partner?	0	1	2	3	4	5
38.	Have you moved house?	0	1	2	3	4	5
39.	Have you had a big problem with your own health?	0	1	2	3	4	5
40.	Have you given birth to any children in the last 5 years?	0	1	2	3	4	5
41.	Have you had serious problems with housing?	0	1	2	3	4	5
42.	Have you had serious dis-agreements with someone close to you (excluding partner)?	0	1	2	3	4	5
43.	Have you been treated by a doctor for a mental or an emotional problem?	0	1	2	3	5	5
44.	Has your partner been treated by a doctor for a mental or an emotional problem?	0	1	2	3	4	5

NOW WE WANT TO ASK YOU SOME QUESTIONS ABOUT YOUR FRIENDS AND RELATIVES.

PLEASE CIRCLE ONE NUMBER FOR EACH QUESTION.

45. How many close friends do you have?

More than 15	1
11 - 15	2
6 - 10	3
3 - 5	4
1 - 2	5
None	6

46. How many of your relatives do you see regularly?
(NOT COUNTING YOUR PARTNER AND CHILDREN)

More than 15	1
11 - 15	2
6 - 10	3
3 - 5	4
1 - 2	5
None	6

47. To how many relatives do you feel really close?
(NOT COUNTING YOUR PARTNER AND CHILDREN)

More than 15	1
11 - 15	2
6 - 10	3
3 - 5	4
1 - 2	5
None	6

48. When you have to make an important decision about such things as e.g. finances, work or moving house, how many people are there who could help you make such a decision?
(NOT COUNTING YOUR PARTNER AND CHILDREN)

Five or more	1
Four	2
Three	3
Two	4
One	5
None	6

49. If you had a serious health problem and needed to get some help around the house, how many people do you know who would help you?
(NOT COUNTING YOUR PARTNER AND CHILDREN)

Five or more	1
Four	2
Three	3
Two	4
One	5
None	6

50. Has the number of people you can turn to for help or support changed in the last 5 years?

There are less people	1
There are the same number	2
There are more people	3

AT TIMES THERE ARE ARGUMENTS AND UPSETS IN EVERY RELATIONSHIP. HOW WELL DO THE FOLLOWING STATEMENTS DESCRIBE THE RELATIONSHIP BETWEEN YOU AND YOUR PARTNER?

PLEASE CIRCLE ONE NUMBER FOR EACH STATEMENT.

		No partner	All the time	Most of the time	Some of the time	Rarely	Never
51.	In general, would you say that things between you and your partner are going well?	1	2	3	4	5	6
52.	How often do you think about divorce, separa-tion or terminating your relationship?	1	2	3	4	5	6
53.	How often do you or your partner leave the house after a fight?	1	2	3	4	5	6
54.	Do you find it easy to confide in your partner?	1	2	3	4	5	6
55.	Do you ever regret that you married (or are living together)?	1	2	3	4	5	6
56.	How often do you and your partner quarrel?	1	2	3	4	5	6
57.	How often do you and your partner "get on each other's nerves"?	1	2	3	4	5	6
58.	When you have an argument with your partner, does the child know about it?	1	2	3	4	5	6
59.	If you have an argument ith your partner, is the child involved?	1	2	3	4	5	6

60. How satisfied are you with your relationship with your husband/partner?
Would you say you are: (please circle one number)

No husband/partner	1
Very satisfied	2
Satisfied	3
Dissatisfied	4
Very dissatisfied	5

NOW WE HAVE SOME QUESTIONS ABOUT HOW YOU BRING UP YOUR CHILD.
THE STATEMENTS BELOW REFER TO YOUR CHILD BORN FIVE YEARS AGO.

PLEASE CIRCLE ONE NUMBER FOR EACH STATEMENT.

		All the time	Most of the time	Some of the time	Rarely	Never
61.	I supervise my child's activities very carefully.	1	2	3	4	5
62.	I expect my child to do as he/she is told without an explanation.	1	2	3	4	5
63.	I watch everything my child does.	1	2	3	4	5
64.	I encourage my child to go outside and play with other children.	1	2	3	4	5
65.	I expect my child to dis-agree with me if he/she thinks I am wrong.	1	2	3	4	5
66.	I encourage my child to do his/her "own thing".	1	2	3	4	5
67.	My child should do as he/ she is told immediately.	1	2	3	4	5
68.	I am too busy to comfort my child.	1	2	3	4	5
69.	I like to cuddle my child.	1	2	3	4	5
70.	Strict discipline is good for my child's later development.	1	2	3	4	5

BELOW ARE LISTED FIVE SITUATIONS.
IF YOUR 5-YEAR-OLD CHILD WERE IN ONE OF THESE SITUATIONS HOW WOULD YOU REACT?

CIRCLE A NUMBER 1 TO 3 FOR SMACKING AND EXPLAINING AND TAKING SOMETHING AWAY.

		Always	Sometimes	Never
	YOUR CHILD REFUSES TO CLEAN UP ROOM			
	Would you:			
71.	Smack the child.	1	2	3
72.	Explain this behaviour is making you feel upset.	1	2	3
73.	Take away something he/she enjoys.	1	2	3
	YOUR CHILD TAKES SOMETHING THAT BELONGS TO ANOTHER CHILD AND PUNCHES HIM/HER			
74.	Smack the child.	1	2	3
75.	Explain this behaviour is making you feel upset.	1	2	3
76.	Take away something he/she enjoys.	1	2	3
	YOUR CHILD MAKES FUN OF A CRIPPLED PERSON			
77.	Smack the child.	1	2	3
78.	Explain this behaviour is making you feel upset.	1	2	3
79.	Take away something he/she enjoys.	1	2	3
	YOUR CHILD TOUCHES A HOT STOVE			
80.	Smack the child.	1	2	3
81.	Explain this behaviour is making you feel upset.	1	2	3
82.	Take away something he/she enjoys.	1	2	3
	YOUR CHILD BREAKS SOMETHING VALUABLE INDOORS AFTER BEING TOLD TO PLAY OUTDOORS			
83.	Smack the child.	1	2	3
84.	Explain this behaviour is making you feel upset.	1	2	3
85.	Take away something he/she enjoys.	1	2	3

AT WHAT AGE WOULD YOU ALLOW YOUR CHILD TO: Age (in years)
(write "0" if never)

86. Travel on a bus alone [ ][ ]

87. Go to the movies with a friend [ ][ ]

88. Go on a holiday with a group of friends of the same age (unsupervised) [ ][ ]

89. Smoke cigarettes [ ][ ]

90. Stay alone in the house while you are away [ ][ ]

91. Drink alcohol [ ][ ]

PLEASE CIRCLE ONE NUMBER FOR EACH QUESTION FROM 92 - 96.

92. In the last week, how often did you smoke cigarettes?

Every day	1
Every few days	2
Once or so only	3
Did not smoke at all	4

93. In the last week, how many cigarettes did you usually smoke per day?

50 or more per day	1
30 – 49 per day	2
20 – 29 per day	3
10 – 19 per day	4
1 – 9 per day	5
Nil smoked	6

94. How often do you drink alcohol?

Daily	1
A few times a week	2
A few times a month	3
A few times a year	4
Rarely	5
Never	6

95. How much alcohol do you usually drink at those times?

Seven or more glasses	1
Five or six glasses	2
Three or four glasses	3
One or two glasses	4
Less than one glass	5
Never drink	6

96. How often did you use cannabis, marihuana, pot in the last month?

Every day	1
Every few days	2
Once or so	3
Not in the last month	4
Never	5

NOW SOME QUESTIONS ABOUT YOUR BACKGROUND.

97. At what level did your partner complete his education?
(please circle one number)

Not applicable (no partner)	1
Opportunity school	2
Primary school	3
Started secondary school	4
Completed Grade 10 - (age 15-16 years)	5
Completed Grade 12 - (age 17-18 years)	6
College (e.g. business, trade, secretarial, teachers')	7
University	8
Other (please specify)	9

____

98. Is your partner presently:
(please circle one number)

Not applicable (no partner)	1
Fully employed	2
Self-employed	3
Employed part-time	4
Unemployed	5
On invalid pension	6
Student	7
Other (please specify)	8

____

99. Are you presently: (please circle one number)

Fully employed	1
Self-employed	2
Employed part-time	3
Unemployed	4
On pension, e.g. invalid, widow's, supporting mother's	5
Student	6
Housewife	7
Other (please specify)	

____

100. What work do or did you usually do for a living? (Please state in detail; for example, if clerk - what type of clerk?)

____
______________________________ [| |] [|]

101. What work does your partner usually do for a living? (Please state in detail. If no partner, write "0".)

____
______________________________ [| |] [|]

	NO	YES
	(please circle one number)	
102. Since the birth of your child 5 years ago, have you ever been employed?	1	2
103. Have you been unemployed at any time in the last 5 years?	1	2
104. Has your partner been unemployed at any time in the last 5 years?	1	2
105. Did you go back to the work you had been doing before the birth of your 5-year-old child?	1	2

106. How long after the birth of your 5-year-old did you wait before you looked for a job? (write "0" if you have not looked for a job)

.... years .... months

107. How long did it take you to get a job once you decided to look for work? (write "0" if you have not looked for a job; write "99" if you still don't have a job)

.... weeks .... days

108 Which of the following best describes who looks after your 5-year-old in your home? (please circle one number)

I do it all	1
I do it with a little help from my partner	2
We look after the child equally	3
My partner does it mainly, and I help a little	4
My partner does it all	5

109. How much time does your 5-year-old spend at pre-school or child care?

.... hours per day (write "0" if not at pre-school)

.... days per week

110. For how long has your 5-year-old been at pre-school or child care?

.... months (write "0" if not at pre-school)

111. In total, how much do you spend each week on child care, pre-school or family day care?

.... dollars (write "0" if nothing spent)

		NO	YES
112.	Are you pregnant now?	1	2

PLEASE CIRCLE ONE NUMBER FOR EACH QUESTION FROM BELOW.

113. What is your present marital status?

Single	1
Living together	2
Married	3
Separated/divorced	4
Widowed	5
Other (please specify)	6

____

114. On the list below, could you circle the number closest to YOUR WHOLE FAMILY'S INCOME (gross) including spouse's income, child endowment, etc. If unsure, circle the number closest to the amount you think may be correct

Per year	Per week	
$0 - $5,199	$ 0 - $99	1
$ 5,200 - $10,399	$100 - $199	2
$10,400 - $15,599	$200 - $299	3
$15,600 - $20,799	$300 - $399	4
$20,800 - $25,999	$400 - $499	5
$26,000 - $31,148	$500 - $599	6
$31,149 or more	$600 or more	7

115. How often do you go to church?

Weekly	1
Monthly	2
Less than once a month	3
Never	4

116. Do you live in a:

House (detached, terrace, town)	1
Flat/unit	2
Share part of a house or flat	3
Caravan, shed, mobile home	4
Hostel	5
Other (please specify)	6

____

117. What is your husband or partner's age?
(if no partner, write "0")

.... years

118. To which of the following groups does your partner belong?

Australian Aborigine	1
Maori/Islander	2
Asian	3
White	4
Other (please specify)	5

____

	NO	YES
	(please circle one number)	
119. Have you ever driven a car within an hour of drinking 3 or more glasses of alcohol?	1	2
120. Has your partner ever driven a car within an hour of drinking 3 or more glasses of alcohol?	1	2
121. Have you ever been arrested for any offences?	1	2

IF YES:

How many times? .... ☐

For what offences? ....…….......……….…....... ☐

.................................…………………………............ ☐

122. Has your partner ever been arrested for any offences?
(please circle one number)

No	1
Yes	2
No partner	3

IF YES:

How many times? .... ☐

For what offences? ....…………………............... ☐

..........................………………………….................. ☐

123. How many times have you been detained in prison?
(please circle one number)

Never	1
Once	2
More than once	3

124. How many times has your partner been detained in prison?
(please circle one number)

Never	1
Once	2
More than once	3

	NO	YES
125. Do you live alone?	1	2
126. Do you live with your partner? (if no partner, please circle "1")	1	2
