# Supplementary material for: Effect of maternal smoking in pregnancy and childhood on child and adolescent sleep outcomes to 21 years: a birth cohort study
Source: BMC Pediatr. 2019 Mar 6;19:70. doi: 10.1186/s12887-019-1439-1 (PMC6402153; doi:10.1186/s12887-019-1439-1)
Supplement: Supplementary file 5 — MUSP maternal 14 years. (PDF 532 kb) [file 12887_2019_1439_MOESM5_ESM.pdf]

MUSP Maternal 14 years

THE QUESTIONS BELOW REFER TO YOUR CHILD BORN AT THE MATER HOSPITAL ON …………./………../…………

PHASE: | 6 | 2 |

CODE: _______________

DATE: _______________

WHICH BEST DESCRIBES YOUR CHILD IN THE LAST SIX MONTHS?

(Circle one number for each question 1-111)

		**Often**	**Sometimes**	**Rarely/ Never**
1	Acts too young for his/her age	1	2	3
2	Allergy	1	2	3
3	Argues a lot	1	2	3
4	Asthma	1	2	3
5	Behaves like opposite sex	1	2	3
6	Bowel movements outside toilet	1	2	3
7	Bragging, boasting	1	2	3
8	Can't concentrate, can't pay attention for long	1	2	3
9	Can't get his/her mind off certain thoughts; obsessions	1	2	3
10	Can't sit still, restless, or hyperactive	1	2	3
11	Clings to adults or too dependent	1	2	3
12	Complains of loneliness	1	2	3
13	Confused or seems to be in a fog	1	2	3

14	Cries a lot	1	2	3
15	Cruel to animals	1	2	3

		(Circle one number)		
		**Often**	**Sometimes**	**Rarely/ Never**
16	Cruelty, bullying, or meanness to others	1	2	3
17	Day-dreams or gets lost in his/her thoughts	1	2	3
18	Deliberately harms self or attempts suicide	1	2	3
19	Demands a lot of attention	1	2	3
20	Destroys his/her own things	1	2	3
21	Destroys things belonging to his/her family or other children	1	2	3
22	Disobedient at home	1	2	3
23	Disobedient at school	1	2	3
24	Doesn't eat well	1	2	3
25	Doesn't get along with other children	1	2	3
26	Doesn't seem to feel guilty after misbehaving	1	2	3
27	Easily jealous	1	2	3
28	Eats or drinks things that are not food - don't include sweets	1	2	3
29	Fears certain animals, situations, or places, other than school	1	2	3
30	Fears going to school	1	2	3
31	Fears he/she might think or do something bad	1	2	3
32	Feels he/she has to be perfect	1	2	3

		(Circle one number)		
		**Often**	**Sometimes**	**Rarely/ Never**
33	Feels or complains that no one loves him/her	1	2	3
34	Feels others are out to get him/her	1	2	3
35	Feels worthless or inferior	1	2	3
36	Gets hurt a lot, accident prone	1	2	3
37	Gets in many fights	1	2	3
38	Gets teased a lot	1	2	3
39	Hangs around with children who get in trouble	1	2	3
40	Hears sounds or voices that aren't there	1	2	3
41	Impulsive or acts without thinking	1	2	3
42	Likes to be alone	1	2	3
43	Lying or cheating	1	2	3
44	Bites fingernails	1	2	3
45	Nervous, highstrung, or tense	1	2	3
46	Nervous movements or twitching	1	2	3
47	Nightmares	1	2	3
48	Not liked by other children	1	2	3
49	Constipated, doesn't move bowels	1	2	3

		(Circle one number)		
		**Often**	**Sometimes**	**Rarely/ Never**
50	Too fearful or anxious	1	2	3
51	Feels dizzy	1	2	3
52	Feels too guilty	1	2	3
53	Overeating	1	2	3
54	Overtired	1	2	3
55	Overweight	1	2	3
56	Physically attacks people	1	2	3
57	Picks nose, skin or other parts of body	1	2	3
58	Plays with own sex parts in public	1	2	3
59	Plays with own sex parts too much	1	2	3
60	Poor school work	1	2	3
61	Poorly coordinated or clumsy	1	2	3
62	Prefers being with older kids	1	2	3
63	Prefers being with younger kids	1	2	3
64	Refuses to talk	1	2	3
65	Repeats certain acts over and over	1	2	3
66	Runs away from home	1	2	3

(Circle one number)

		Often	Sometimes	Rarely/ Never
67	Screams a lot	1	2	3
68	Secretive, keeps things to self	1	2	3
69	Sees things that aren’t there	1	2	3
70	Self-conscious or easily embarrassed	1	2	3
71	Sets fires	1	2	3
72	Sexual problems	1	2	3
73	Showing off or clowning	1	2	3
74	Shy or timid	1	2	3
75	Sleeps less than most kids	1	2	3
76	Sleeps more than most kids during day and/or night	1	2	3
77	Smears or plays with bowel movements	1	2	3
78	Speech problem	1	2	3
79	Stares blankly	1	2	3
80	Steals at home	1	2	3
81	Steals outside home	1	2	3
82	Stores up things he/she doesn’t need	1	2	3
83	Strange behaviour	1	2	3

		(Circle one number)		
		**Often**	**Sometimes**	**Rarely/ Never**
84	Strange ideas	1	2	3
85	Stubborn, sullen, or irritable	1	2	3
86	Sudden changes in mood or feelings	1	2	3
87	Sulks a lot	1	2	3
88	Suspicious	1	2	3
89	Swearing or obscene language	1	2	3
90	Talks about killing self	1	2	3
91	Talks or walks in sleep	1	2	3
92	Talks too much	1	2	3
93	Teases a lot	1	2	3
94	Temper tantrums or hot temper	1	2	3
95	Thinks about sex too much	1	2	3
96	Threatens people	1	2	3
97	Thumb-sucking	1	2	3
98	Too concerned with neatness or cleanliness	1	2	3
99	Trouble sleeping	1	2	3
100	Truancy, skips school	1	2	3

(Circle one number)

		Often	Sometimes	Rarely/ Never
101	Underactive, slow moving, or lacks energy	1	2	3
102	Unhappy, sad or depressed	1	2	3
103	Unusually loud	1	2	3
104	Uses alcohol or drugs for nonmedical purposes	1	2	3
105	Vandalism	1	2	3
106	Wets self during the day	1	2	3
107	Wets the bed	1	2	3
108	Whining	1	2	3
109	Wishes to be of the opposite sex	1	2	3
110	Withdrawn, doesn't get involved with others	1	2	3
111	Worries	1	2	3

NOW WE HAVE SOME QUESTIONS ABOUT IOW YOU COMMUNICATE WITH YOUR CHILD .
HOW WELL DO THE FOLLOWING STATEMENTS DESCRIBE WHAT HAPPENS IN YOUR HOME?

(Please circle one number for each question 112-131)

		Strongly Agree	Agree	Unsure	Disagree	Strongly Disagree
112	I can discuss my beliefs with my child without feeling restrained or embarrassed.	1	2	3	4	5
113	Sometimes I have trouble believing everything my child tells me.	1	2	3	4	5
114	My child is always a good listener.	1	2	3	4	5
115	I am sometimes afraid to ask my child for what I want.	1	2	3	4	5
116	My child has a tendency to say things to me which would be better left unsaid.	1	2	3	4	5
117	My child can tell how I'm feeling without asking.	1	2	3	4	5
118	I am very satisfied with how my child and I talk together.	1	2	3	4	5
119	If I were in trouble, I could tell my child.	1	2	3	4	5
120	I openly show affection to my child.	1	2	3	4	5
121	When we are having a problem, I often give my child the silent treatment.	1	2	3	4	5
122	I am careful about what I say to my child.	1	2	3	4	5
123	When talking with my child, I have a tendency to say things that would be better left unsaid.	1	2	3	4	5

		Strongly Agree	Agree	Unsure	Disagree	Strongly Disagree
124	When I ask questions, I get honest answers from my child.	1	2	3	4	5
125	My child tries to understand my point of view.	1	2	3	4	5
126	There are topics I avoid discussing with my child.	1	2	3	4	5
127	I find it easy to discuss problems with my child.	1	2	3	4	5
128	It is very easy for me to express all my true feelings to my child.	1	2	3	4	5
129	My child nags/bothers me	1	2	3	4	5
130	My child insults me when he/she is angry with me.	1	2	3	4	5
131	I don't think I can tell my child how I really feel about some things.	1	2	3	4	5

HOW OFTEN HAS YOUR CHILD HAD THE FOLLOWING IN THE **LAST SIX MONTHS**?

(Circle one number for each question 132-138)

		Often	Sometimes	Rarely/ Never
132	Aches or pains (not headaches)	1	2	3
133	Headaches	1	2	3
134	Nausea, feel sick	1	2	3
135	Problems with eyes	1	2	3
136	Rashes or other skin problems	1	2	3
137	Stomach aches or cramps	1	2	3
138	Vomiting, throwing up	1	2	3

HAS YOUR CHILD HAD ANY OF THE FOLLOWING?

(Circle one number; or each question 139-150)

		No	Yes
139	Acne (pimples)	1	2
140	Asthma	1	2
141	Epileptic fits	1	2
142	Diabetes	1	2
143	Food allergies	1	2
144	A head injury with loss of consciousness	1	2
145	Broken bones or fractures	1	2
146	Burns requiring hospitalization	1	2
147	Poisoning requiring hospitalization	1	2
148	In the **last year**, an accident needing a doctor's help	1	2
149	In the **last year**, bedwetting	1	2
150	In the **last year**, snoring	1	2

151 In general, in the **last year**, do you think your child's health has been?

(Circle one number)

Excellent..1
Good...2
Fair 3
Poor 4

152 How many times has your child been to a doctor in the last year?

__________________ times (write 0 if never)

153 What is the total number of times your child has been admitted to hospital since birth?

__________________ times (write 0 if never)

154 Does your child wear glasses or contact leases? **(Circle one number)**

- No 1
- Yes, prescribed by doctor 2
- Yes, but not prescribed by doctor 3

155 Does your child have a hearing problem? **(Circle one number)**

- No 1
- Yes, mild 2
- Yes, uses a hearing aid 3

156 Has your child got holes and/or fillings in his/her teeth?
(Circle one number)

- No 1
- Yes, a few 2
- Yes, a lot 3

157 About how many close friends does your child have?
(Circle one number)

- None 1
- One 2
- Two or three 3
- Four or more 4

158 On average how many hours a day (Monday to Friday) does your child sit and watch TV?
(Circle one number)

- 7 hours or more 1
- 5 - less than 7 hours 2
- 3 - less than 5 hours 3
- 1 - less than 3 hours 4
- Less than 1 hour 5
- Never watches TV 6

159 On average how many hours a day (Saturday to Sunday) does your child sit and watch TV?
(Circle one number)

- 7 hours or more 1
- 5 - less than 7 hours 2
- 3 - less than 5 hours 3
- 1 - less than 3 hours 4
- Less than 1 hour 5
- Never watches TV 6

160 For writing is your child? **(Circle one number)**

Left handed.. 1
Right handed 2
Either handed.. 3

161 What is your child's year level (grade) at school?

_______________________ year level

162 How many primary schools did your child attend?

_______________________ number of schools

HAS YOUR CHILD EVER HAD CONTACT WITH ANY OF THE FOLLOWING?

(Circle one number for each question 163-165)

		**No**	**Yes**
163	Guidance Officer	1	2
164	Children's Services	1	2
165	Police or Juvenile Aid	1	2

166 Has your child ever been suspended from school for any reason?
(Circle one number)

No.. 1
Yes.. 2

167 How would you describe your child's current school performance overall?
(Circle one number)

Below average 1
A bit below average... 2
Average 3
A bit above average... 4
Above average... 5

(Circle one number for each question 168-170)

		No	Yes
168	Has your child ever repeated a year at school?	1	2
169	Has your child ever needed remedial help?	1	2
170	Has your child ever attended a special education unit or special school for learning difficulties?	1	2

171 How important is it to you for your child to do well at school?

(Circle one number)

Not important 1
Quite important 2
Fairly important.. 3
Very important 4

172 What would you most like your child to do after he/she finishes school?

(Circle one number)

Get a job 1
Trade/Apprenticeship 2
College 3
University... 4
Other (please specify)...................................... 5

NOW WE HAVE SOME QUESTIONS ABOUT YOURSELF.
HAVE ANY OF THE FOLLOWING HAPPENED TO YOU IN THE **LAST SEVEN YEARS**?

(Circle one number for each time this happened in questions 173-188)

			Yes - it happened				
		**No**	**1 yr. ago**	**2 yrs. ago**	**3 yrs. ago**	**4 yrs. ago**	**5-7 yrs. ago**
173	Have you lost your job?	0	1	2	3	4	5
174	Has your partner lost his job?	0	1	2	3	4	5
175	Have you or your partner had a problem with the police?	0	1	2	3	4	5
176	Has anyone close to you died?	0	1	2	3	4	5
177	Have you been divorced?	0	1	2	3	4	5
178	Have you and your partner separated?	0	1	2	3	4	5
179	Have you changed partners?	0	1	2	3	4	5
180	Have you had trouble finding somewhere to live?	0	1	2	3	4	5

			Yes - it happened					
		**No**	**1 yr. ago**	**2 yrs. ago**	**3 yrs. ago**	**4 yrs. ago**	**5-7 yrs. ago**	
181	Have you been treated for a mental or an emotional problem?	0	1	2	3	4	5	☐☐
182	Has your partner been treated for a mental or an emotional problem?	0	1	2	3	4	5	☐☐
183	Have you had any serious financial problems?	0	1	2	3	4	5	☐☐
184	Have you had a serious illness or injury?	0	1	2	3	4	5	☐☐
185	Has your current partner had a serious illness or injury?	0	1	2	3	4	5	☐☐
186	Have you changed your address?	0	1	2	3	4	5	☐☐
187	Have you experienced violence in a relationship with a partner?	0	1	2	3	4	5	☐☐
188	Have you had some other major crisis or worry?	0	1	2	3	4	5	☐☐

189 In general, in the last year, do you think your health has been?

(Circle one number)

Excellent.. 1
Good... 2
Fair 3
Poor 4

190 How many times have you been to a doctor about your own health in the **last six months**?

__________________ times (write 0 if never) [ ][ ]

191 To what extent has your physical health or emotional problems interfered with your normal activities in the **last six months**?

(Circle one number)

Not at all.. 1
Slightly 2
Moderately 3
Quite a bit... 4
Extremely 5

HAVE YOU BEEN TO ANY OF THE FOLLOWING FOR A MENTAL OR EMOTIONAL PROBLEM IN THE **LAST SEVEN YEARS**?

(Circle one number for each question 192-195)

		**No**	**Yes**
192	A natural therapist	1	2
193	A general practitioner	1	2
194	A counsellor, psychologist or psychiatrist	1	2
195	Hospital for admission	1	2

196 In the last week, how many cigarettes did you usually smoke per day?
(Circle one number)

50 or more per day 1
30 - 49 per day... 2
20 - 29 per day... 3
10 - 19 per day... 4
1 - 9 per day... 5
None 6

197 Do you smoke? **(Circle one number)**

Nonsmoker 1
Inside the house.. 2
Outside the house 3
Both 4

198 Does your partner smoke? **(Circle one number)**

Non-smoker.. 1
Inside the house.. 2
Outside the house 3
Both 4
No partner.. 5

199 How often do you drink alcohol? **(Circle one number)**

Daily.. 1
A few times a week 2
A few times a month 3
A few times a year.. 4
Rarely 5
Never 6

200 How much alcohol do you usually drink at those times?
(Circle one number)

Seven or more glasses 1
Five or six glasses 2
Three or four glasses 3
One or two glasses.. 4
Less than one glass... 5
Never drink... 6

NOW WE ARE INTERESTED IN HOW YOU HAVE BEEN FEELING RECENTLY.

(Circle one number for each question 201-214)

		**All the time**	**Most of the time**	**Some of the time**	**Rarely**	**Never**
201	I have worried about every little thing	1	2	3	4	5
202	I have been so miserable that I have had difficulty sleeping	1	2	3	4	5
203	I have been breathless or had a pounding of my heart	1	2	3	4	5
204	I have been so worked up that I couldn't sit still	1	2	3	4	5
205	I have been depressed without knowing why	1	2	3	4	5
206	I have gone to bed not caring if I never woke up	1	2	3	4	5
207	For no good reason I have had feelings of panic	1	2	3	4	5
208	I have been so low in spirit that I have sat up for ages doing absolutely nothing	1	2	3	4	5
209	I have had a pain or tense feeling in my neck or head	1	2	3	4	5
210	The future seems hopeless	1	2	3	4	5
211	Worrying has kept me awake at night	1	2	3	4	5
212	I have lost interest in just about everything	1	2	3	4	5
213	I have been so anxious that I couldn't make up my mind about the simplest thing	1	2	3	4	5
214	I have been so depressed that I have thought of doing away with myself	1	2	3	4	5

AT TIMES THERE ARE ARGUMENTS AND UPSETS IN EVERY RELATIONSHIP. HOW WELL DO THE FOLLOWING STATEMENTS DESCRIBE THE RELATIONSHIP BETWEEN YOU AND YOUR PARTNER?

(Circle one number far each question 215-223)

		**No partner**	**All the time**	**Most of the time**	**Some of the time**	**Rarely**	**Never**
215	In general, would you say that things between you and your partner are going well?	1	2	3	4	5	6
216	How often do you think about divorce, separation or terminating your relationship?	1	2	3	4	5	6
217	How often do you or your partner leave the house after a fight?	1	2	3	4	5	6
218	Do you find it easy to confide in your partner?	1	2	3	4	5	6
219	Do you ever regret that you married (or are living together)?	1	2	3	4	5	6
220	How often do you and your partner quarrel?	1	2	3	4	5	6
221	How often do you and your partner ‘get on each other's nerves'?	1	2	3	4	5	6
222	When you have an argument with your partner, does the child know about it?	1	2	3	4	5	6
223	If you have an argument with your partner, is the child involved?	1	2	3	4	5	6

224 How satisfied are you with your relationship with your husband/partner?

(Circle one number)

No husband/partner 1
Very satisfied.. 2
Satisfied... 3
Very dissatisfied.. 4
Dissatisfied... 5

NO MATTER HOW WELL A COUPLE GET ALONG, THERE ARE TIMES WHEN THEY DISAGREE.
If you had a disagreement, has your partner ever done any of the following in the **last year**?
If no partner write 0 ....

(Circle one number for each question 225-231)

		Often	Sometimes	Never
225	Argued heatedly without yelling	1	2	3
226	Yelled at and/or insulted you	1	2	3
227	Sulked and/or refused to talk about the problem	1	2	3
228	Threw something at you	1	2	3
229	Pushed, grabbed or shoved you	1	2	3
230	Tried to hit you	1	2	3
231	Hit you	1	2	3

DUE TO DISAGREEMENT WITH A PARTNER IN THE **LAST SEVEN YEARS**?
If no partner write 0 ....

(Circle one number for each question 232-234)

		No	Yes
232	Have you been physically hurt?	1	2
233	Have you needed medical attention?	1	2
234	Have you called the police?	1	2

235 On the list below, could you circle the number closest to **your whole family's income** (gross) including spouse's income, child endowment, etc. If unsure, circle the number closest to the amount you think may be correct.

**Per year**		**Per week**	
$0 - $10,399	...	$0 - $199	1
$10,400 - $15,599	...	$200 - $299	2
$15,600 - $20,799	...	$300 - $399	3
$20,800 - $25,999	...	$400 - $499	4
$26,000 - $31,148	...	$500 - $599	5
$31,149 - $36,349	...	$600 - $699	6
$36,350 or more	...	$700 or more	7

236 What is your present marital status?

(Circle one number)

Single (never married)...................................... 1
Living together 2
Married 3
Separated/divorced... 4
Widowed 5
Other (please specify)....................................... 6

237 To which of the following groups does your partner belong?

(Circle one number)

No partner.. 1
Australian Aboriginal....................................... 2
Maori/Islander 3
Asian.. 4
White 5
Other (please specify)....................................... 6

238 Do you live with a partner?

(Circle one number)

No 1
Yes... 2

239 How long has your present relationship lasted? …………

If not in a relationship write 0 ………

240 Is your present partner the father of this child?

(Circle one number)

No 1
Yes... 2
No partner.. 3

241 What is **your** relationship to this child?

(Circle one number)

Mother 1
Father... 2
Stepmother 3
Stepfather 4
Other (Please specify) 5

242 Does this child live with you?

(Circle one number)

All the time.. 1
Most of the time 2
About half the time.. 3
Less than half the time 4
Never 5

243 How many years has this child lived with you?

___________________ years

244 IF YOU ARE **NOT** THIS CHILD'S MOTHER, DOES THE CHILD LIVE WITH HIS/HER MOTHER?

(Circle one number)

Not applicable, I am the mother........................ 1
All the time.. 2
Most of the time 3
About half the time.. 4
Less than half the time 5
Never 6

245 How many years has this child lived with his/her mother?

______________________ years

246 How many times has this child lived with someone else as the main care giver for over three months?

______________________ times

WRITE THE NUMBER OF CHILDREN WHO USUALLY LIVE WITH YOU:

		**Number of Children**
247	**Your** children from your present relationship	
248	**Your** children from a previous relationship	
249	Stepchildren eg your partner's children	
250	Other children eg foster children, niece, nephew	

251 Are you presently:

(Circle one number)

Fully employed	1
Self-employed	2
Employed part-time (temporary)	3
Unemployed	4
On pension	5
Student	6
Home duties	7
Other (please specify)	8

252 Is your partner presently:

If no partner write 0 …………… **(Circle one number)**

Fully employed	1
Self-employed	2
Employed part-time (temporary)	3
Unemployed	4
On pension	5
Student	6
Home duties	7
Other (please specify)	8

253 To which of the following occupational groups do you belong?

(Circle one number)

Home duties/not presently employed	1
Labourers and related workers	2
Tradesperson; Clerks Salespersons and Personal Service Workers; Plant and Machine Operators and Drivers	3
Managers and Administrators; Professionals; Para-Professionals	4

254 To which of the following occupational groups does your partner belong?

If no partner write 0 ....

(Circle one number)

Home duties/not presently employed 1
Labourers and related workers 2
Tradesperson; Clerks; Salespersons and Personal Service
Workers; Plant and Machine Operators and Drivers................ 3
Managers and Administrators; Professionals;
Para-Professionals 4

HOW MUCH ARE THE FOLLOWING A PROBLEM IN THE AREA WHERE YOU LIVE?

(Circle one number for each question 255-263)

		**Major problem**	**Moderate problem**	**Small problem**	**No problem**
255	Vandalism/graffiti	1	2	3	4
256	House burglaries	1	2	3	4
257	Car stealing	1	2	3	4
258	Drug abuse	1	2	3	4
259	Violence in the streets	1	2	3	4
260	Unemployment	1	2	3	4
261	Noisy and/or reckless driving	1	2	3	4
262	Alcohol abuse	1	2	3	4
263	School truancy	1	2	3	4

NOW WE HAVE SOME QUESTIONS ABOUT **FOOD**.

264 How often does your family sit down to eat a meal together?

(Circle one number)

At least once a day 1
A few times a week 2
About once a week 3
Less than once a week 4

265 How important to you is it that your family eats together?

(Circle one number)

Not really important 1
Quite important 2
Very important 3

266 How often does your family go out to eat?

(Circle one number)

About once a week 1
Once or twice a month 2
Several times a year 3
Rarely or never 4

267 How often does your family get takeaway food?

(Circle one number)

Once or more a week 1
A few times a month 2
Less than once a month 3
Rarely or never 4

268 Who decides what food will be bought each week?

(Circle one number)

I decide completely 1
I decide mostly 2
My partner and I decide equally 3
My partner decides mostly 4
My partner decides completely 5

HOW OFTEN DO YOU HAVE THE FOLLOWING?

(Circle one number for each question 269-277)

		Once or more a day	Most days	2 or 3 times a week	Rarely or never
269	Sweets/lollies	1	2	3	4
270	Fresh fruit	1	2	3	4
271	Fast food (pizza, burgers, chips)	1	2	3	4
272	Cooked vegetables	1	2	3	4
273	Salad	1	2	3	4
274	Red meat	1	2	3	4
275	Soft drink/cordial	1	2	3	4
276	Fruit juice	1	2	3	4
277	Cakes/biscuits	1	2	3	4

HOW IMPORTANT ARE THE FOLLOWING WHEN YOU CHOOSE FOOD?

(Circle one number for each question 278-284)

		**Not Important**	**Quite Important**	**Very Important**
278	Taste of the food	1	2	3
279	Whether my children will eat it	1	2	3
280	Whether my partner will eat it	1	2	3
281	Whether it is easy to prepare	1	2	3
282	Whether it is healthy	1	2	3
283	Whether it is fattening	1	2	3
284	Whether it is expensive	1	2	3
