# Supplementary material for: Effect of maternal smoking in pregnancy and childhood on child and adolescent sleep outcomes to 21 years: a birth cohort study
Source: BMC Pediatr. 2019 Mar 6;19:70. doi: 10.1186/s12887-019-1439-1 (PMC6402153; doi:10.1186/s12887-019-1439-1)
Supplement: Supplementary file 6 — MUSP youth 14 years. (PDF 143 kb) [file 12887_2019_1439_MOESM6_ESM.pdf]

MUSP Youth 14 Years

PLEASE WRITE YOUR DATE OF BIRTH ..../........./19....... **PHASE:** | 6 | 1 |

CODE: ____________

DATE: ____________

WHICH BEST DESCRIBES YOUR BEHAVIOUR IN THE **LAST SIX MONTHS**?

(Circle one number for each question 1-112)

		**Often**	**Sometimes**	**Rarely/ Never**
1	I act too young for my age	1	2	3
2	I have an allergy	1	2	3
3	I argue a lot	1	2	3
4	I have asthma	1	2	3
5	I act like the opposite sex	1	2	3
6	I like animals	1	2	3
7	I brag	1	2	3
8	I have trouble concentrating or paying attention	1	2	3
9	I can't get my mind off certain thoughts	1	2	3
10	I have trouble sitting still	1	2	3
11	I am too dependent on adults	1	2	3
12	I feel lonely	1	2	3
13	I feel confused or in a fog	1	2	3
14	I cry a lot	1	2	3

15	I am pretty honest	1	2	3

		(Circle one number)		
		**Often**	**Sometimes**	**Rarely/ Never**
16	I am mean to others	1	2	3
17	I day-dream a lot	1	2	3
18	I deliberately try to hurt or kill myself	1	2	3
19	I try to get a lot of attention	1	2	3
20	I destroy my own things	1	2	3
21	I destroy things belonging to others	1	2	3
22	I disobey my parents	1	2	3
23	I disobey at school	1	2	3
24	I don't eat as well as I should	1	2	3
25	I don't get along with other kids	1	2	3
26	I don't feel guilty after doing something I shouldn't	1	2	3
27	I am jealous of others	1	2	3
28	I am willing to help others when they need help	1	2	3
29	I am afraid of certain animals, situations, or places, other than school	1	2	3
30	I am afraid of going to school	1	2	3
31	I am afraid I might think or do something bad	1	2	3
32	I feel that I have to be perfect	1	2	3

		(Circle one number)		
		**Often**	**Sometimes**	**Rarely/ Never**
33	I feel that no one loves me	1	2	3
34	I feel that others are out to get me	1	2	3
35	I feel worthless or inferior	1	2	3
36	I accidentally get hurt a lot	1	2	3
37	I get in many fights	1	2	3
38	I get teased a lot	1	2	3
39	I hang around with kids who get in trouble	1	2	3
40	I hear sounds or voices that other people think aren't there	1	2	3
41	I act without stopping to think	1	2	3
42	I would rather be alone than with others	1	2	3
43	I lie or cheat	1	2	3
44	I bite my fingernails	1	2	3
45	I am nervous or tense	1	2	3
46	Parts of my body twitch or make nervous movements	1	2	3
47	I have nightmares	1	2	3
48	I am not liked by other kids	1	2	3
49	I can do certain things better than most kids	1	2	3

		(Circle one number)		
		**Often**	**Sometimes**	**Rarely/ Never**
50	I am fearful or anxious	1	2	3
51	I feel dizzy	1	2	3
52	I feel too guilty	1	2	3
53	I eat too much	1	2	3
54	I feel overtired	1	2	3
55	I am overweight	1	2	3
56	I physically attack people	1	2	3
57	I pick my skin or other parts of my body	1	2	3
58	I can be pretty friendly	1	2	3
59	I like to try new things	1	2	3
60	My school work is poor	1	2	3
61	I am poorly coordinated or clumsy	1	2	3
62	I would rather be with older kids than with kids my own age	1	2	3
63	I would rather be with younger kids than with kids my own age	1	2	3
64	I refuse to talk	1	2	3
65	I repeat certain actions over and over	1	2	3
66	I run away from home	1	2	3

(Circle one number)

		**Often**	**Sometimes**	**Rarely/ Never**
67	I scream a lot	1	2	3
68	I am secretive or keep things to myself	1	2	3
69	I see things that other people think aren't there	1	2	3
70	I am self-conscious or easily embarrassed	1	2	3
71	I set fires	1	2	3
72	I can work well with my hands	1	2	3
73	I show off or clown	1	2	3
74	I am shy	1	2	3
75	I sleep less than most kids	1	2	3
76	I sleep more than most kids during day and/or night	1	2	3
77	I have a good imagination	1	2	3
78	I have a speech problem	1	2	3
79	I stand up for my rights	1	2	3
80	I steal at home	1	2	3
81	I steal from places other than home	1	2	3
82	I store up things I don't need	1	2	3
83	I do things other people think are strange	1	2	3

		(Circle one number)		
		**Often**	**Sometimes**	**Rarely/ Never**
84	I have thoughts that other people would think are strange	1	2	3
85	I am stubborn	1	2	3
86	My moods or feelings change suddenly	1	2	3
87	I enjoy being with other people	1	2	3
88	I am suspicious	1	2	3
89	I swear or use dirty language	1	2	3
90	I think about killing myself	1	2	3
91	I like to make others laugh	1	2	3
92	I talk too much	1	2	3
93	I tease others a lot	1	2	3
94	I have a hot temper	1	2	3
95	I think about sex too much	1	2	3
96	I threaten to hurt people	1	2	3
97	I like to help others	1	2	3
98	I am too concerned about being neat and clean	1	2	3
99	I have trouble sleeping	1	2	3
100	I cut classes or skip school	1	2	3

		(Circle one number)		
		**Often**	**Sometimes**	**Rarely/ Never**
101	I don't have much energy	1	2	3
102	I am unhappy, sad or depressed	1	2	3
103	I am louder than other kids	1	2	3
104	I use alcohol or drugs for nonmedical purposes	1	2	3
105	I try to be fair to others	1	2	3
106	I enjoy a good joke	1	2	3
107	I try to take life easy	1	2	3
108	I try to help other people when I can	1	2	3
109	I wish I were of the opposite sex	1	2	3
110	I keep from getting involved with others	1	2	3
111	I worry a lot	1	2	3
112	I smoke cigarettes	1	2	3

How often have you had the following in the **last six months?**

(Circle one number for each question 113-119)

	**Often**	**Sometimes**	**Rarely/ Never**
113 Aches or pains (not headaches)	1	2	3
114 Headaches	1	2	3
115 Nausea, feel sick	1	2	3
116 Problems with eyes	1	2	3
117 Rashes or other skin problems	1	2	3
118 Stomach aches or cramps	1	2	3
119 Vomiting, throwing up	1	2	3

120 On average how many **hours a day (Monday to Friday)** do you sit and watch TV?
(Circle one number)

7 hours or more 1
5 - less than 7 hours........................ 2
3 - less than 5 hours........................ 3
1 - less than 3 hours........................ 4
Less than 1 hour 5
Never watch TV 6

121 On average how many **hours a day (Saturday to Sunday)** do you sit and watch TV?
(Circle one number)

7 hours or more 1
5 - less than 7 hours........................ 2
3 - less than 5 hours........................ 3
1 - less than 3 hours........................ 4
Less than 1 hour 5
Never watch TV 6

122 In the **last week** how often did you smoke cigarettes?

(Circle one number)

Every day	1
Every few days	2
Once or so only	3
Did not smoke at all	4

123 In the last week how many cigarettes did you smoke?

(Circle one number)

50 or more	1
30-49	2
20-29 .....:.....................	3
10-19	4
1-9............................	5
Nil smoked	6

124 How often do you drink alcohol?

(Circle one number)

Daily	1
A few times a week	2
A few times a month..................	3
A few times a year	4
Rarely	5
Never	6

125 How much alcohol do you usually drink at those times?

(Circle one number)

Seven or more glasses	1
Five or six glasses	2
Three or four glasses	3
One or two glasses	4
Less than one glass	5
Never drink	6

126 For writing, which hand do you use?

(Circle one number)

Left hand 1
Right hand... 2
Either hand 3

127 For most tasks which hand do you use?

(Circle one number)

Always right.. 1
Usually right... 2
Always left 3
Usually left... 4
Either hand 5

128 In general, over the last year, do you think your health has been?

(Circle one number)

Excellent... 1
Good... 2
Fair 3
Poor 4

129 Do you have anyone you can talk to about your problems?

(Circle one number)

No.. 1
Yes 2

130 How often did you exercise or play sports in the last week?

(Circle one number)

Not at all... 1
1 day 2
2 or 3 days.. 3
4 or 5 days.. 4
6 or 7 days.. 5

131 How are you doing at school in English?

(Circle one number)

Below average... 1
A bit below average 2
Average 3
A bit above average....................................... 4
Above average... 5

132 How are you doing at school in Maths?

(Circle one number)

Below average... 1
A bit below average 2
Average 3
A bit above average....................................... 4
Above average... 5

133 How are you doing at school in Science?

(Circle one number)

Below average... 1
A bit below average 2
Average 3
A bit above average....................................... 4
Above average... 5

134 How are you doing at school overall?

(Circle one number)

Below average... 1
A bit below average 2
Average 3
A bit above average....................................... 4
Above average... 5

135 How important do your friends think it is to work hard at school?

(Circle one number

Not important...	1
A bit important...	2
Fairly important..	3
Very important	4

136 Is the amount of effort you put into your school work?

(Circle one number)

Below average..	1
A bit below average	2
Average	3
A bit above average......................................	4
Above average..	5

137 How important do you think it is for you to do well at school?

(Circle one number)

Not important...	1
A bit important...	2
Fairly important..	3
Very important	4

138 How important does your mother think it is for you to work hard at school?

(Circle one number)

No mother	1
Not important...	2
A bit important...	3
Fairly important..	4
Very important	5

139 How important does your father think it is for you to work hard at school?

(Circle one number)

No father 1
Not important 2
A bit important 3
Fairly important.. 4
Very important 5

140 What would you like to do when you finish school?

(Circle one number)

Get a job 1
Trade/apprenticeship 2
College 3
University... 4
Other (please specify) 5

141 What would you like to be when you leave school (eg plumber, hairdresser)?

____

____

____
