# Supplementary material for: Effect of maternal smoking in pregnancy and childhood on child and adolescent sleep outcomes to 21 years: a birth cohort study
Source: BMC Pediatr. 2019 Mar 6;19:70. doi: 10.1186/s12887-019-1439-1 (PMC6402153; doi:10.1186/s12887-019-1439-1)
Supplement: Supplementary file 7 — MUSP youth 21 years. (PDF 968 kb) [file 12887_2019_1439_MOESM7_ESM.pdf]

STRICTLY CONFIDENTIAL

MUSP Youth 21 Years

7	1

CODE A: | | | | | | |

DATE: | | | | | | | | |

MATER-UNIVERSITY OF QUEENSLAND STUDY OF PREGNANCY AND OUTCOMES

School of Social Science

YOUNG ADULT QUESTIONNAIRE

(MUSP21)

PLEASE NOTE: ***ALL*** **ANSWERS ARE** ***STRICTLY CONFIDENTIAL.***
ANSWERING EACH QUESTION IS VOLUNTARY.
THERE ARE NO RIGHT OR WRONG ANSWERS OR TRICK QUESTIONS.

If you feel the need to discuss any issues that might arise as a result of your participation in this study, please use the contact list of counselling services attached to the back of this questionnaire. Please detach this list before returning your completed questionnaire.

Thank you very much for agreeing to participate in this study.

PLEASE CIRCLE ONE NUMBER FOR EACH QUESTION *ONLY*, UNLESS SPECIFIED OTHERWISE.

PLEASE CIRCLE ONE NUMBER FOR EACH QUESTION

Please turn the page to begin.

PLEASE CIRCLE ONE NUMBER FOR EACH QUESTION

Male 1 **Female** 2

YOUR BACKGROUND

1. What is your date of birth?

(e.g. | *10* | *6* | *1982* | *)*

Day	Month	Year

2. In the LAST WEEK did you have a full-time or part-time job of any kind?

- Yes, worked full-time for payment or profit………. 1
- Yes, worked part-time for payment or profit……… 2
- Yes, but absent on holidays, on paid leave, on strike or temporarily stood down…………………… 3
- Yes, unpaid work in a family business…………….. 4
- Yes, other unpaid work……………………………… 5
- No, did not have a job……………………………….. 6

3. In your main job held LAST WEEK, were you?

- A wage or salary earner…………………………….. 1
- Conducting own business with employees………. 2
- Conducting own business without employees……. 3
- A helper not receiving wages………………………. 4
- No job…………………………………………………. 5

4. What is/was your main occupation (*choose ONE option only*)?

- **Manager or Administrator** ……………………………………………… 1
  (e.g. personnel manager, managing supervisor)
- **Professional**…………………………………………………………………….. 2
  (e.g. teacher, social worker, doctor, artist)
- **Paraprofessional** …………………………………………………………… 3
  (e.g. welfare worker, technical officer, police)
- **Trade** ……………………………………………………………………………….. 4
  (e.g. hairdresser, cook, mechanic)
- **Administrative assistant** ……………………………………………………... 5
  (e.g. secretary, telephonist)
- **Sales and personal service worker** ………………………………………… 6
  (e.g. sales assistant, bar attendant, child care worker, enrolled nurse)
- **Machine operator or driver** …………………………………………………... 7
  (e.g. sewing machinist)
- **Manual worker** ……………………………………………………………….. 8
  (e.g. labourer, cleaner, kitchen hand)
- **Student**………………………………………………………………………… 9
- **Never had a paid job**…………………………………………………………... 10
- **Other** *(Please specify on line)*________________________________ 11

PLEASE CIRCLE ONE NUMBER FOR EACH QUESTION

5. How many hours do you normally do in all your PAID jobs each week?

No paid job………….. 1
1 – 15 hours……….. 2
16 – 24 hours……….. 3
25 – 34 hours……….. 4
35 – 40 hours……….. 5
41- 48 hours……….. 6
49 hours more……… 7

6. From the list below, please circle the number closest to your WEEKLY INCOME (gross ie. *before* tax or other deductions) including payments from Centrelink, etc. if unsure, circle the number closest to the amount you think may be correct.

Nil Income per week………	………………………………………...	1
$1 - $39 per week ………...	($1 - $2,079 per year)…………………	2
$40 - $79 per week………..	($2,080 - $4,159 per year)…………..	3
$80 - $119 per week………	($4,160- $6,239 per year)………….....	4
$120 - $159 per week…….	($6,240 - $8,319 per year)…………..	5
$160 - $199 per week…….	($8,320 - $10,399 per year)…………..	6
$200 - $299 per week…….	($10,400 - $15,599 per year)………..	7
$300 - $399 per week…….	($15,600 - $20,799 per year)………..	8
$400 - $499 per week…….	($20,800 - $25,999 per year)………..	9
$500 - $599 per week…….	($26,000 - $31,199 per year)………..	10
$600 - $699 per week…….	($31,200 - $36,399 per year)………..	11
$700 - $799 per week…….	($36,400 - $41,599 per year)………..	12
$800 or more per week…..	($41,600 or more per year)…………...	13

7. Have you personally been receiving any of the following benefits in the LAST 6 MONTHS?

No benefit received……………………………. 1
Youth Allowance………………………………. 2
Austudy…………………………………………. 3
Newstart Allowance…………………………… 4
Disability Support Pension……………………. 5
Carer Pension………………………………….. 6
Sickness Allowance…………………………… 7
Parenting Payment……………………………. 8
Other *(Please specify on line)*
______________________________________ 9

8. Are you attending a school or any other educational institution?

No……………………. 1
Yes, full-time……….. 2
Yes, part-time……….. 3

9. What type of educational institution are you attending?

Not attending any educational institution…………….. 1
A university or other higher educational institution….. 2
A technical or further educational institution
(including TAFE colleges)……………………………… 3
A secondary school…………………………………….. 4
Some other educational institution
(please specify on line) _______________________ 5

PLEASE CIRCLE ONE NUMBER FOR EACH QUESTION

10. What is the highest level of education you have completed?

Primary school…………………………………………… 1
Started secondary school………………………………. 2
Completed secondary school………………………….. 3
College (eg. Business, Trade, Secretarial, Teachers)…. 4
TAFE………………………………………………………… 5
University…………………………………………………. 6
Other
(please specify on line) ________________________ 7

11. In which year did you complete this?
(eg. 1999)

12. What was your OP score for Year 12?
(If no OP score, write 0)

13. Do you live in a:

House (detached/semi/terrace/ town house)……………………… 1
Flat/Unit……………………………………………………………... 2
Caravan, Shed, Tent, Mobile home, Temporary dwelling, Boat… 3
Hostel………………………………………………………………... 4
Other *(please specify on line)* ______________________________ 5

14. Do you:

Live at home with parents………………………………………………………. 1
Own your accommodation outright…………………………………………… 2
Live in own accommodation with loan or mortgage…………………………… 3
Rent your own accommodation………………………………………………... 4
Other *(please specify on line)* ____ 5

15. At what age did you first leave your family home?
(If never left home, write 0)

		years old

16. Do you live alone?

No……….. 1
Yes……… 2

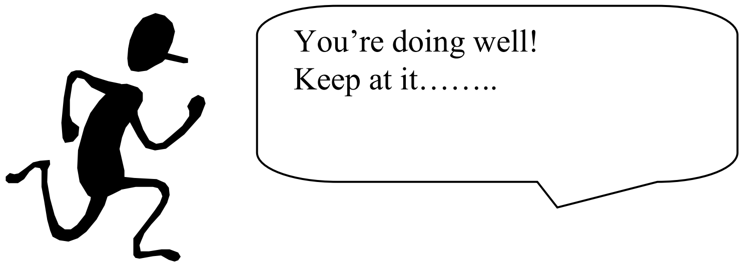

PLEASE CIRCLE ONE NUMBER FOR EACH QUESTION

How much are the following a problem in the area where you live?	Don't know	No problem	Small problem	Moderate problem	Major problem
**17.** Vandalism/Graffiti	1	2	3	4	5
**18.** House burglaries	1	2	3	4	5
**19.** Car stealing	1	2	3	4	5
**20.** Drug abuse	1	2	3	4	5
**21.** Violence in the streets	1	2	3	4	5
**22.** Unemployment	1	2	3	4	5
**23.** Noisy and/or reckless driving	1	2	3	4	5
**24.** Alcohol abuse	1	2	3	4	5
**25.** School truancy ("wagging" school)	1	2	3	4	5

26. During the PAST MONTH, how many days did you feel unsafe in your neighbourhood?

0 days………………….. 1
1 day…………………… 2
2 – 3 days…………….. 3
4 or more days……….. 4

27. In the PAST 12 MONTHS, how many times have you been a victim of crime?
(If none, write 0)

☐ times

28. What is your present marital status?

Never married……………………………………. 1
Living together (de facto)……………………….. 2
Married………………………………………..………. 3
Separated but not divorced……………………….. 4
Divorced…………………………………………….. 5
Widowed…………………………………………… 6

29. Do you live with your partner (*ie. spouse, de facto, boyfriend, or girlfriend*)?

No partner……………….. 1
Do not live with partner… 2
Yes……………………….. 3

30. Do you currently have a boyfriend or girlfriend?

No………….. 1
Yes……….. 2

31. How long has your present relationship lasted?
(If not in a relationship, write 0. Please fill in appropriate box according to whether relationship has lasted a period of years, OR months, OR weeks, OR days)

A Years		B Months		C Weeks		D Days
☐	**or**	☐	**or**	☐	**or**	☐

PLEASE CIRCLE ONE NUMBER FOR EACH QUESTION

32. To which of the following groups does YOUR PARTNER belong?

No partner…………………………………………………	1
Australian Aboriginal……………………………………..	2
Torres Strait Islander…………………………………….	3
Australian South Sea Islander…………………………..	4
Maori………………………………………………………	5
Asian………………………………………………………	6
White………………………………………………………	7
Other *(Please specify on line)*__________________	8

YOUR CHILDREN

		Number
33.	How many of your biological children are currently **IN YOUR CARE?** *(Write 0, if none)*	
34.	How many of your biological children are **NOT** in your care? *(Write 0, if none)*	

35. Please provide the birth dates of your biological children below:

(eg | *01* | *05* | *1999* | *)*

36. What is the date of birth of your **first-born** biological child? *(Write 0, if none)*

Day	Month	Year

37. What is the date of birth of your **second-born** biological child? *(Write 0, if none)*

Day	Month	Year

38. What is the date of birth of your **third-born** biological child? *(Write 0, if none)*

Day	Month	Year

LIFESTYLE

39. Which of the following best describes your smoking status now?

I have never smoked…………	1
I used to smoke………………..	2
I now smoke occasionally…….	3
I now smoke regularly………..	4

40. If you USED to smoke, how long ago did you quit smoking?

Never smoked ………………………………	1
Within the last six months…………………..	2
6 – 12 months ago…………………………....	3
More than one year ago……………….………	4
I still smoke……………………………………	5

PLEASE CIRCLE ONE NUMBER FOR EACH QUESTION

41. How many times have you tried to quit in the LAST 6 MONTHS?

Didn't smoke in the last 6 months...……	1
Never tried to quit………………………..	2
Once………………………………………	3
Twice……………………………………...	4
Three times……………………………….	5
More than 3 times………………………..	6

42. At what age did you start smoking?
(If never smoked write 0)

☐☐ years old

43. In the LAST WEEK, how many cigarettes did you usually smoke PER DAY?
(*Circle one number only)*

Did not smoke at all……….	1
1-9 per day…………………	2
10-19 per day……………...	3
20 – 29 per day…………..	4
30 – 49 per day…………..	5
50 or more per day………..	6

44. At what age did you START drinking alcohol (*ie. more than just a few sips*)?
(If never started, write 0)

☐☐ years old

45. How often do you drink alcohol?
(Circle one number only)

Never drink…………………	1
Daily………………………...	2
A few times a week……….	3
A few times a month………	4
A few times a year………...	5
Rarely………………………	6

46. How much alcohol do you usually drink at those times?
(Circle one number only)

Never drink…………………	1
Less than one glass ……...	2
One or two glasses ……….	3
Three or four glasses……..	4
Five or six glasses………...	5
Seven or more glasses…...	6

PLEASE CIRCLE ONE NUMBER FOR EACH QUESTION

**To what extent has ALCOHOL impacted on your life? Circle the response which most closely applies to you over the PAST 4 WEEKS:**		**Have never drunk alcohol**	**Not at all**	**Mildly**	**Moderately**	**Severely**
**47.**	I am troubled about my alcohol use	1	2	3	4	5
**48.**	My alcohol use has limited my performance at work, school or other activities	1	2	3	4	5
**49.**	I have worried about my present or future health because of my alcohol use	1	2	3	4	5
**50.**	I have been limited in going to certain places because of my alcohol use	1	2	3	4	5
**51.**	I have felt frustrated with myself because of my alcohol use	1	2	3	4	5
**52.**	I have felt that my alcohol use is controlling my life	1	2	3	4	5
**53.**	Using alcohol has interfered with my social life	1	2	3	4	5
**54.**	I have felt that using alcohol is preventing me from achieving what I want in life	1	2	3	4	5

Now we are going to ask you about different drugs you may have used. As with all questions, any information you provide will be kept STRICTLY CONFIDENTIAL.

55. In the LAST MONTH, how often did you use cannabis, marijuana, pot etc.?
(Circle one number only)

Have never used these... 1
Every day………………... 2
Every few days………….. 3
Once or so………………. 4
Not in the last month…… 5

56. At what age did you start using cannabis?
(If never write 0)

[ ][ ] years old

57. Have you ever injected illegal drugs?

No……… 1
Yes…….. 2

58. Have you injected illegal drugs in the LAST 12 MONTHS?

No……… 1
Yes…….. 2

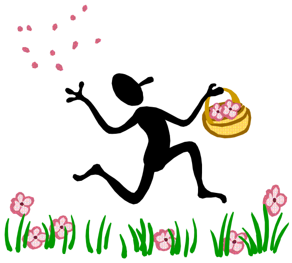

PLEASE CIRCLE ONE NUMBER FOR EACH QUESTION

During the LAST 12 MONTHS how often have you used the following?		Never used	Not used in the past year	A few times during the year	A few times a month	A few times a week
**59.**	Amphetamines like speed, uppers, or pep pills	1	2	3	4	5
**60.**	Ecstasy or other designer drugs	1	2	3	4	5
**61.**	Inhalants like glue, paint, petrol, amyl nitrate, nitrous oxide ("bulbs")	1	2	3	4	5
**62.**	Cocaine	1	2	3	4	5
**63.**	Heroin	1	2	3	4	5
**64.**	Hallucinogens like acid, LSD or magic mushrooms	1	2	3	4	5

				NON-MEDICAL USE			
During the LAST 12 MONTHS how often have you used the following for NON-MEDICAL purposes?		Used only for medical purposes	Have never used at all	Not used during the past year	A few times during the year	A few times a month	A few times a week
**65.**	Ritalin or Dexedrine (ADHD medication)	1	2	3	4	5	6
**66.**	Pain killers like panadol and codeine	1	2	3	4	5	6
**67.**	Ventolin	1	2	3	4	5	6
**68.**	Steroids	1	2	3	4	5	6
**69.**	Sedatives like sleeping pills, barbiturates or downers	1	2	3	4	5	6
**70.**	Tranquilizers like valium, rohypnol or mogodon	1	2	3	4	5	6

To what extent has DRUG/SUBSTANCE USE impacted on your life? Circle the response which most closely applies to you over the PAST 4 WEEKS:		Have not used	Not at all	Mildly	Moderately	Severely
**71.**	I am troubled about my use	1	2	3	4	5
**72.**	My use has limited my performance at work, school or other activities	1	2	3	4	5
**73.**	I have worried about my present or future health because of my use	1	2	3	4	5
**74.**	I have been limited in going to certain places because I use	1	2	3	4	5
**75.**	I have felt frustrated with myself because of my use	1	2	3	4	5
**76.**	I have felt that my use is controlling my life	1	2	3	4	5
**77.**	Using has interfered with my social life	1	2	3	4	5
**78.**	I have felt that using is preventing me from achieving what I want in life	1	2	3	4	5

PLEASE CIRCLE ONE NUMBER FOR EACH QUESTION

WELL-BEING

Below is a list of the ways you might have felt or behaved. How often have you felt this way during the PAST WEEK?:		Rarely or none of the time (less than 1 day)	Some or a little of the time (1-2 days)	Occasionally or a moderate amount of the time (3-4 days)	Most or all of the time (5-7 days)
**79.**	I was bothered by things that usually don't bother me	1	2	3	4
**80.**	I did not feel like eating: my appetite was poor	1	2	3	4
**81.**	I felt that I could not shake off the blues even with help from my family or friends	1	2	3	4
**82.**	I felt that I was just as good as other people	1	2	3	4
**83.**	I had trouble keeping my mind on what I was doing	1	2	3	4
**84.**	I felt depressed	1	2	3	4
**85.**	I felt that everything I did was an effort	1	2	3	4
**86.**	I felt hopeful about the future	1	2	3	4
**87.**	I thought my life had been a failure	1	2	3	4
**88.**	I felt fearful	1	2	3	4
**89.**	My sleep was restless	1	2	3	4
**90.**	I was happy	1	2	3	4
**91.**	I talked less than usual	1	2	3	4
**92.**	I felt lonely	1	2	3	4
**93.**	People were unfriendly	1	2	3	4
**94.**	I enjoyed life	1	2	3	4
**95.**	I had crying spells	1	2	3	4
**96.**	I felt sad	1	2	3	4
**97.**	I felt that people disliked me	1	2	3	4
**98.**	I could not get 'going'	1	2	3	4

PLEASE CIRCLE ONE NUMBER FOR EACH QUESTION

99. How satisfied are you with your life as a whole these days? What would you say you are?

Very satisfied………….. 1
Satisfied……………….. 2
Dissatisfied……………. 3
Very dissatisfied………. 4

100. How would you say you feel these days? Would you say you are:

Very happy………... 1
Fairly happy………. 2
Not too happy…….. 3
Very unhappy……... 4

DRIVING

101. At what age did you first start to drive on a public road?
(If you do not drive, write 0)

[ ][ ] years

102. At what age did you first obtain your learner's permit?
(If you have not obtained a permit, write 0)

[ ][ ] years

103. At what age did you first obtain your driver's licence?
(If you don't have a license, write 0)

[ ][ ] years

104. How many hours PER WEEK do you drive?
(If you don't drive, write 0)

[ ][ ] hours per week

105. How many traffic accidents have you been involved in when you have been the driver of the vehicle (*including accidents off road eg. car parks*)?
(If you don't drive, write 0)

[ ][ ] accidents

Which of the following do you drive on the road?	No	Yes
**106.** Car	1	2
**107.** Motorbike	1	2

108. What is the approximate value of the vehicle you drive?

Don't drive………………………..	1
0 - $2,500…………………………	2
$2,501 - $5,000…………………	3
$5,001-$10,000…………………	4
$10,001-$20,000………………..	5
$20,001-$30,000………………..	6
Over $30,001……………………	7
Don't know………………………..	8

109. How skilful do you think you are as a driver?

Not applicable………..	1
Very skilled……………	2
Skilled…………………..	3
Unskilled………………	4
Very unskilled…………	5

**Please indicate whether you as a driver, have EVER done ANY of the following:**		**Don't drive**	**No**	**Yes**
**110.**	Speeding	1	2	3
**111.**	Driven an unsafe or un-roadworthy vehicle	1	2	3
**112.**	Ignored red traffic lights	1	2	3
**113.**	Ignored a police signal, order or direction	1	2	3
**114.**	Ignored a stop or give way sign	1	2	3
**115.**	Failed to give way, other than by ignoring a traffic sign	1	2	3
**116.**	Failed to keep left	1	2	3
**117.**	Failed to wear helmet, seat belt or restraint	1	2	3
**118.**	Improper turns	1	2	3
**119.**	Ignored traffic lane arrows in roundabout	1	2	3
**120.**	Other *(please specify):* ______________________	1	2	3

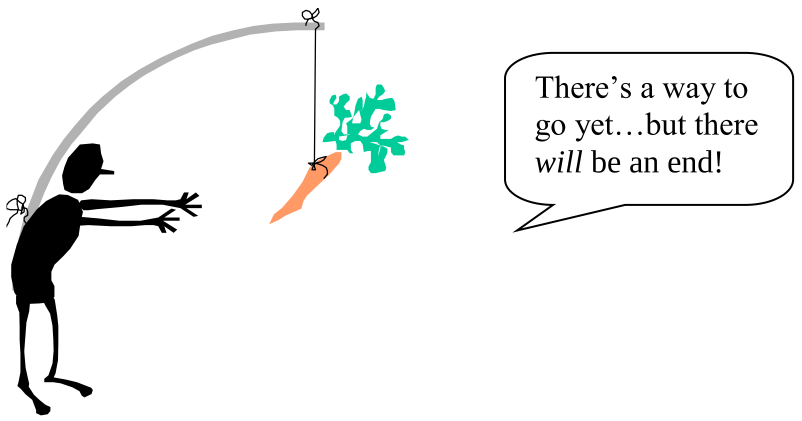

PLEASE CIRCLE ONE NUMBER FOR EACH QUESTION

**Have you EVER been fined or charged for ANY of the following?**		**Don't drive**	**No**	**Yes**
**121.**	Speeding	1	2	3
**122.**	Driving an unsafe or un-roadworthy vehicle	1	2	3
**123.**	Ignoring red traffic lights	1	2	3
**124.**	Ignoring a police signal, order or direction	1	2	3
**125.**	Ignoring a stop or give way sign	1	2	3
**126.**	Failing to give way, other than by ignoring a traffic sign	1	2	3
**127.**	Failing to keep left	1	2	3
**128.**	Failing to wear helmet, seat belt or restraint	1	2	3
**129.**	Improper turns	1	2	3
**130.**	Ignoring traffic lane arrows in roundabout	1	2	3
**131.**	Other *(please specify)*: ____________________	1	2	3

**How often do you do the following?**		**Never**	**Hardly ever**	**Occasionally**	**Quite often**	**Frequently**	**Nearly all the time**
**132.**	Tail-gate another car to try and make its driver go faster or get out of the way	1	2	3	4	5	6
**133.**	Run red lights	1	2	3	4	5	6
**134.**	Ignore the speed limits	1	2	3	4	5	6
**135.**	Indicate your hostility to a driver who annoys you by whatever means you can	1	2	3	4	5	6
**136.**	Become impatient with a slow driver in the outer lane and overtake on the inside (left) lane	1	2	3	4	5	6
**137.**	Drive even though you may be over the legal blood-alcohol limit	1	2	3	4	5	6
**138.**	Drive after using illegal drugs	1	2	3	4	5	6
**139.**	Not wear a seat belt or helmet	1	2	3	4	5	6
**140.**	Overtake another vehicle over double lines	1	2	3	4	5	6
**141.**	Drive while tired	1	2	3	4	5	6
**142.**	Get involved with unofficial 'races' with other drivers	1	2	3	4	5	6
**143.**	Give chase to a driver with the intention of giving him/her a piece of your mind	1	2	3	4	5	6

144. Have you ever had your driver's licence restricted, cancelled, suspended, or gone to court to appeal for your licence?

No…….. 1
Yes…… 2

**In the PAST 12 MONTHS, have you:**		**No**	**Yes**
**145.**	Driven an unregistered car or motorbike?	1	2
**146.**	Driven a car or motorbike without a licence?	1	2
**147.**	Taken someone else's car or motorbike without asking their permission?	1	2

**How often have you experienced the following when being a PASSENGER?**		**Never**	**Rarely**	**Some-times**	**Often**
**148.**	The driver speeds	1	2	3	4
**149.**	The driver is drunk	1	2	3	4
**150.**	The driver does not wear a seat belt	1	2	3	4
**151.**	The driver overtakes other vehicles over double lines	1	2	3	4
**152.**	The driver is tired while driving	1	2	3	4

PHYSICAL EXERCISE

The next few questions are concerned with your average physical activity over the LAST 6 MONTHS.

153. On average, how many times a week do you exercise vigorously for a period of at least 20 minutes *('Vigorously' means exercise which makes you breathe harder or puff and pant, and includes such activities as swimming, tennis, netball, athletics and running)?*

Not at all…………………. 1
1 or 2 times a week…….. 2
3 or more times a week... 3

154. For how long have you been doing this level of activity?

Less than 3 months……………………………… 1
More than 3 months but less than 1 year……... 2
More than 1 year but less than 5 years……….. 3
5 years or more………………………………….. 4

155. On average, how many times a week do you engage in less vigorous exercise for recreation, sport or health and fitness purposes, which does not make you breath harder or puff or pant. This includes activities such as bike riding, dancing, etc.?

Not at all…………………. 1
1 or 2 times a week…….. 2
3 or more times a week... 3

156. On average, how many times a week do you walk for recreation or exercise?

None at all………………. 1
1 or 2 times a week…….. 2
3 or more times a week... 3

The next few questions are about the PAST 2 WEEKS ONLY.

157. In the PAST 2 WEEKS, did you engage in vigorous exercise *(eg. vigorous sports such as football, netball, tennis, squash, athletics, jogging, or running, keep-fit exercises, vigorous swimming*)?

No… 1
Yes.. 2

158. How many sessions of vigorous exercise did you have over the 2 WEEK period?
(If no exercise, write 0)

[ ] sessions

159. Please estimate the TOTAL TIME exercising vigorously during the PAST 2 WEEKS?
(If no exercise, write 0)

[ ] hours [ ] minutes

160. In the PAST 2 WEEKS, did you engage in less vigorous exercise for recreation, sport or health-fitness purposes which did not make you breathe harder or puff and pant?

No… 1
Yes.. 2

161. How many sessions of less vigorous exercise did you have over the 2 WEEK period?

[ ] sessions

162. In the PAST 2 WEEKS, did you walk for recreation or exercise?

No… 1
Yes.. 2

163. How many times did you walk for recreation or exercise?

[ ] times

164. In the PAST 2 WEEKS, did you engage in vigorous activity, apart from exercise, which made you breathe harder or puff or pant *(eg. carrying loads, heavy gardening, chopping wood, labouring- at home, during employment or anywhere else)*?

No… 1
Yes.. 2

165. How many sessions of these types of vigorous activity did you have over the 2 WEEK period?

[ ] sessions

PLEASE CIRCLE ONE NUMBER FOR EACH QUESTION

166. Please estimate the TOTAL TIME you engaged in vigorous activities, apart from exercise, during the PAST 2 WEEKS?

[ ] hours [ ] minutes

FAT INTAKE

How many times PER WEEK do you do the following?:	Never or hardly ever	Less than once per week	Once or twice a week	Three to five times per week	Six or more times per week
**167.** Eat fried food with a batter or breadcrumb coating	1	2	3	4	5
**168.** Eat gravy, cream sauces or cheese sauce	1	2	3	4	5
**169.** Add butter, margarine, oil or sour cream to vegetables, cooked rice or spaghetti	1	2	3	4	5
**170.** Eat vegetables that are fried or roasted with fat or oil (this will include stir fry's)	1	2	3	4	5
**171.** Eat sausages, Devon, salamis, meat pies, hamburgers or bacon	1	2	3	4	5
**172.** Eat chips or French fries	1	2	3	4	5
**173.** Eat pastries, cakes, sweet biscuits or croissants	1	2	3	4	5
**174.** Eat chocolate, chocolate biscuits or sweet snack bars	1	2	3	4	5
**175.** Eat potato crisps, corn chips or nuts	1	2	3	4	5
**176.** Eat cream	1	2	3	4	5
**177.** Eat ice cream	1	2	3	4	5
**178.** Eat cheddar, edam or other hard cheese, cream cheese, or cheese like camembert	1	2	3	4	5

179. How is your meat usually cooked?

Fried………………………………………….... 1
Stewed or goulash………………………….... 2
Grilled or roasted with added fat or oil…….. 3
Grilled or roasted without added fat or oil… 4
Eat meat occasionally or never……………. 5

180. How do you spread butter/margarine on your bread?

Thickly……………………………… 1
Medium…………………………….. 2
Thinly……………………………….. 3
Don't use butter or margarine…… 4

181. What type of milk do you drink or use in cooking or tea and coffee?

Condensed milk…………….. 1
Full-cream…………………… 2
Full-cream and reduced fat... 3
Reduced fat…………………. 4
Skim or none………………… 5

182. How much of the skin on your chicken do you eat?

Most or all of the skin…... 1
Some of the skin………... 2
None of the skin………… 3
I am a vegetarian………. 4

183. How much of the fat on your meat do you eat?

Most or all of the fat….. 1
Some of the fat……….. 2
None of the fat………… 3
I am a vegetarian…….. 4

YOUR HEALTH

Have you EVER been told by a doctor that you have any of the following (including during pregnancy)?	No	Yes
**184.** Diabetes (high blood sugars)	1	2
**185.** Hypertension (high blood pressure)	1	2
**186.** Eczema	1	2
**187.** Asthma	1	2
**188.** Depression	1	2
**189.** Anxiety disorder	1	2
**190.** Autism (Autistic Spectrum Disorder/Asperger's Syndrome)	1	2
**191.** Schizophrenia	1	2
**192.** Migraine	1	2
**193.** Tension headache	1	2
**194.** Attention Deficit (Hyperactivity) Disorder	1	2
**195.** Liver disease	1	2
**196.** Gall bladder disease	1	2
**197.** Obstructive sleep apnoea (severe snoring with night waking)	1	2

198. Do you have any other illness, disability or handicap?

No…………………………………………………………………. 1
Yes *(please describe):* ______________________________ 2

____

PLEASE CIRCLE ONE NUMBER FOR EACH QUESTION

In the LAST 12 MONTHS:		Number
**199.**	How many wheezing attacks have you had?	
**200.**	How many nights has your sleep been disturbed because of wheezing?	

201. **In the LAST 12 MONTHS, have you experienced wheezing that was ever severe enough to limit your speech to only one or two words at a time between breaths?**

No… 1
Yes.. 2

202. **Do you take medication to prevent attacks of asthma?**

No… 1
Yes.. 2

203. **Have you taken asthma medication in the LAST 2 WEEKS?**

No… 1
Yes.. 2

204. **Have you EVER been admitted to hospital because of an asthma attack?**

No… 1
Yes.. 2

How many times have you consulted the following people for your own health in the LAST 12 MONTHS?		Never	Once or twice	Three or four times	Five or six times	Seven or more times
**205.**	Family doctor or another general practitioner	1	2	3	4	5
**206.**	A hospital doctor (e.g.. outpatients or casualty)	1	2	3	4	5
**207.**	A specialist doctor	1	2	3	4	5
**208.**	An allied health professional (e.g.. optician, dentist, physiotherapist, podiatrist, dietician, mental health professional, etc)	1	2	3	4	5
**209.**	An “alternative” health practitioner (e.g.. chiropractor, naturopath, acupuncturist, herbalist, etc)	1	2	3	4	5

210. **How many times have you been admitted to hospital in the LAST 12 MONTHS?**
(Write 0, if nil)

[ ] times

211. **Do you take vitamin or health supplements?**

No … 1
Yes… 2

PLEASE CIRCLE ONE NUMBER FOR EACH QUESTION

SLEEPING

212 . **During the PAST MONTH, in general, how many hours of actual sleep did you get EACH day/night?** (***This may be different than the number of hours you spend in bed.***)

[ ][ ] hours sleep per day

**During the PAST MONTH, how often have the following occurred?**	**Less than once a week**	**Once to twice per week**	**Three or more times a week**
**213.** Waking during the night	1	2	3
**214.** My snoring	1	2	3
**215.** Restlessness in sleep	1	2	3
**216.** Daytime drowsiness	1	2	3

217. **During the PAST MONTH, how would you rate your sleep quality overall?**

Very good……………. 1
Fairly good………….. 2
Fairly bad…………….. 3
Very bad……………... 4

218. **During the PAST MONTH, how often have you taken medicine (prescribed or "over the counter") to help you sleep?**

Not during the past month……. 1
Less than once a week……….. 2
Once or twice a week………… 3
Three or more times a week…. 4

219. **During the PAST MONTH, how often have you had trouble staying awake while driving, eating meals, or engaging in social activity?**

Not during the past month……. 1
Less than once a week……….. 2
Once or twice a week………… 3
Three or more times a week…. 4

220. **During the PAST MONTH, how much of a problem has it been for you to keep up enough enthusiasm to get things done?**

No problem at all…………….. 1
Only a very slight problem….. 2
Somewhat of a problem…….. 3
A very big problem…………... 4

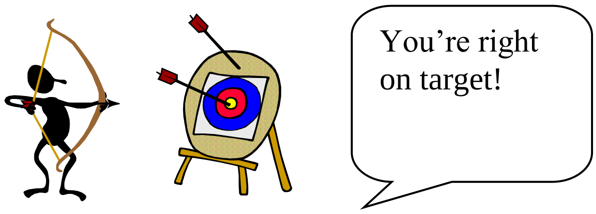

PLEASE CIRCLE ONE NUMBER FOR EACH QUESTION

BEHAVIOUR

**Below is a list of items that describe people. For each item, please circle the response that best describes yourself over the PAST 6 MONTHS (even if some don't seem to apply to you).**		**Not true**	**Somewhat or sometimes true**	**Very true or often true**
**221.**	I act too young for my age	0	1	2
**222.**	I make good use of my opportunities	0	1	2
**223.**	I argue a lot	0	1	2
**224.**	I work up to my ability	0	1	2
**225.**	I act like the opposite sex	0	1	2
**226.**	I use drugs (other than alcohol) for nonmedical purposes	0	1	2
**227.**	I brag	0	1	2
**228.**	I have trouble concentrating or paying attention	0	1	2
**229.**	I can't get my mind off certain thoughts	0	1	2
**230.**	I have trouble sitting still	0	1	2
**231.**	I am too dependent on others	0	1	2
**232.**	I feel lonely	0	1	2
**233.**	I feel confused or in a fog	0	1	2
**234.**	I cry a lot	0	1	2
**235.**	I am pretty honest	0	1	2
**236.**	I am mean to others	0	1	2
**237.**	I daydream a lot	0	1	2
**238.**	I deliberately try to hurt or kill myself	0	1	2
**239.**	I try to get a lot of attention	0	1	2
**240.**	I destroy my things	0	1	2
**241.**	I destroy things belonging to others	0	1	2
**242.**	I worry about my future	0	1	2
**243.**	I break rules at work, where I study, or elsewhere	0	1	2
**244.**	I don't eat as well as I should	0	1	2
**245.**	I don't get along with other people	0	1	2
**246.**	I don't feel guilty after doing something I shouldn't	0	1	2
**247.**	I am jealous of others	0	1	2
**248.**	I get along badly with my family	0	1	2

Copyright T.M. Achenbach Reproduced by permission.

PLEASE CIRCLE ONE NUMBER FOR EACH QUESTION

Over the PAST 6 MONTHS:		Not true	Somewhat or sometimes true	Very true or often true
**249.**	I am afraid of certain animals, situations, or places	0	1	2
**250.**	My relations with the opposite sex are poor	0	1	2
**251.**	I am afraid I might think or do something bad	0	1	2
**252.**	I feel that I have to be perfect	0	1	2
**253.**	I feel that no one loves me	0	1	2
**254.**	I feel that others are out to get me	0	1	2
**255.**	I feel worthless or inferior	0	1	2
**256.**	I accidentally get hurt a lot	0	1	2
**257.**	I get in many fights	0	1	2
**258.**	I get teased a lot	0	1	2
**259.**	I hang around with others who get in trouble	0	1	2
**260.**	I hear sound or voices that other people think aren't there	0	1	2
**261.**	I am impulsive or act without thinking	0	1	2
**262.**	I would rather be alone than with others	0	1	2
**263.**	I lie or cheat	0	1	2
**264.**	I bite my fingernails	0	1	2
**265.**	I am nervous and tense	0	1	2
**266.**	Parts of my body twitch or make nervous movements	0	1	2
**267.**	I lack self-confidence	0	1	2
**268.**	I am not liked by others	0	1	2
**269.**	I can do certain things better than other people	0	1	2
**270.**	I am too fearful or anxious	0	1	2
**271.**	I feel dizzy or lightheaded	0	1	2
**272.**	I feel too guilty	0	1	2
**273.**	I eat too much	0	1	2
**274.**	I feel overtired	0	1	2
**275.**	I am overweight	0	1	2
**276.**	I physically attack people	0	1	2
**277.**	I pick my skin or other parts of my body	0	1	2
**278.**	I fail to finish things I should do	0	1	2

Copyright T.M. Achenbach Reproduced by permission.

PLEASE CIRCLE ONE NUMBER FOR EACH QUESTION

Over the PAST 6 MONTHS:		Not true	Somewhat or sometimes true	Very true or often true
**279.**	There is very little I enjoy	0	1	2
**280.**	My study or job performance is poor	0	1	2
**281.**	I am poorly coordinated or clumsy	0	1	2
**282.**	I would rather be with older people than with people of my own age	0	1	2
**283.**	I would rather be with younger people than with people of my own age	0	1	2
**284.**	I refuse to talk	0	1	2
**285.**	I repeat certain acts over and over	0	1	2
**286.**	I have trouble making or keeping friends	0	1	2
**287.**	I scream or yell a lot	0	1	2
**288.**	I am secretive or keep things to myself	0	1	2
**289.**	I see things that other people think aren't there	0	1	2
**290.**	I am self-conscious or easily embarrassed	0	1	2
**291.**	I set fires	0	1	2
**292.**	I meet my responsibilities to my family	0	1	2
**293.**	I show off or clown	0	1	2
**294.**	I am shy or timid	0	1	2
**295.**	My behaviour is irresponsible	0	1	2
**296.**	I sleep more than most other people during day and/or night	0	1	2
**297.**	I have trouble making decisions	0	1	2
**298.**	I have a speech problem	0	1	2
**299.**	I stand up for my rights	0	1	2
**300.**	I worry about my job or school work	0	1	2
**301.**	I steal	0	1	2
**302.**	I store up too many things I don't need	0	1	2
**303.**	I do things other people think are strange	0	1	2
**304.**	I have thoughts that other people would think are strange	0	1	2
**305.**	I am stubborn, sullen, or irritable	0	1	2
**306.**	My moods or feelings change suddenly	0	1	2
**307.**	I enjoy being with other people	0	1	2
**308.**	I am suspicious	0	1	2

Copyright T.M. Achenbach Reproduced by permission.

PLEASE CIRCLE ONE NUMBER FOR EACH QUESTION

Over the PAST 6 MONTHS:		Not true	Somewhat or sometimes true	Very true or often true
**309.**	I drink too much alcohol or get drunk	0	1	2
**310.**	I think about killing myself	0	1	2
**311.**	I do things that may cause me trouble with the law	0	1	2
**312.**	I talk too much	0	1	2
**313.**	I tease others a lot	0	1	2
**314.**	I have a hot temper	0	1	2
**315.**	I think about sex too much	0	1	2
**316.**	I threaten to hurt people	0	1	2
**317.**	I like to help others	0	1	2
**318.**	I enjoy a good joke	0	1	2
**319.**	I have trouble sleeping	0	1	2
**320.**	I have a good imagination	0	1	2
**321.**	I don’t have much energy	0	1	2
**322.**	I am unhappy, sad, or depressed	0	1	2
**323.**	I am louder than others	0	1	2
**324.**	I like to make others laugh	0	1	2
**325.**	I try to be fair to others	0	1	2
**326.**	I feel that I can’t succeed	0	1	2
**327.**	I like to take life easy	0	1	2
**328.**	I like to try new things	0	1	2
**329.**	I wish I were of the opposite sex	0	1	2
**330.**	I keep from getting involved with others	0	1	2
**331.**	I worry a lot	0	1	2
**332.**	I am too concerned about how I look	0	1	2
**333.**	I fail to pay debts or meet other financial responsibilities	0	1	2
**334.**	I have nightmares	0	1	2
**335.**	I worry about my relations with the opposite sex	0	1	2

Copyright T.M. Achenbach Reproduced by permission.

PLEASE CIRCLE ONE NUMBER FOR EACH QUESTION

**I have the following physical problems WITHOUT a *known medical cause* over the PAST 6 MONTHS:**		Not true	Somewhat or sometimes true	Very true or often true
**336.**	Aches or pains (**not** stomach or headaches)	0	1	2
**337.**	Headaches	0	1	2
**338.**	Nausea, feel sick	0	1	2
**339.**	Problems with eyes (not if corrected by glasses)	0	1	2
**340.**	Rashes or other skin problems	0	1	2
**341.**	Stomach-aches	0	1	2
**342.**	Vomiting, throwing up	0	1	2
**343.**	Heart pounding or racing	0	1	2
**344.**	Numbness or tingling in body parts	0	1	2

Copyright T.M. Achenbach Reproduced by permission.

345. Do you spend money on gambling *(eg. buy lottery tickets, play the pokies, go to the casino, bet on horses, dogs, etc.)*?

No……….. 1
Yes……… 2

346. On average, how much do you spend PER WEEK on gambling activities *(in whole dollars*)?

$ [ ] per week

**In the PAST 12 MONTHS, have you:**		No	Yes
**347.**	Shoplifted	1	2
**348.**	Stolen things or parts from a car or a motorbike	1	2
**349.**	Broken into a house or a building to steal things	1	2
**350.**	Deliberately hurt or beaten up somebody	1	2
**351.**	Been hurt deliberately or beaten up by somebody else	1	2
**352.**	Forced someone to do sexual things when they didn't want to	1	2

353. Have you ever been given a warning by the police *(do not include traffic offences)*?

No… 1
Yes.. 2

354. Have you ever had to go to court because of something you did?

No… 1
Yes.. 2

PLEASE CIRCLE ONE NUMBER FOR EACH QUESTION

BELIEFS AND PRACTICES

How much do you agree with the following statements?		Strongly disagree	Disagree	Unsure	Agree	Strongly Agree
**355.**	I prefer to go to places I know	1	2	3	4	5
**356.**	I avoid things that are dangerous	1	2	3	4	5
**357.**	I like to try new foods	1	2	3	4	5
**358.**	I like to take a gamble	1	2	3	4	5
**359.**	I prefer to be in familiar places	1	2	3	4	5
**360.**	I like to do the unexpected	1	2	3	4	5
**361.**	Without taking risks, life becomes boring	1	2	3	4	5
**362.**	Life is about experiencing the unexpected	1	2	3	4	5
**363.**	I would never try bungee jumping	1	2	3	4	5
**364.**	I prefer to order familiar foods when I eat out	1	2	3	4	5
**365.**	I like the idea of travelling to strange places	1	2	3	4	5
**366.**	I like doing new things	1	2	3	4	5
**367.**	I like the idea of trying new things at least once	1	2	3	4	5
**368.**	If you don't take chances, you don't enjoy life	1	2	3	4	5
**369.**	I enjoy the idea of taking a risk	1	2	3	4	5
**370.**	Why take chances when you don't need to	1	2	3	4	5

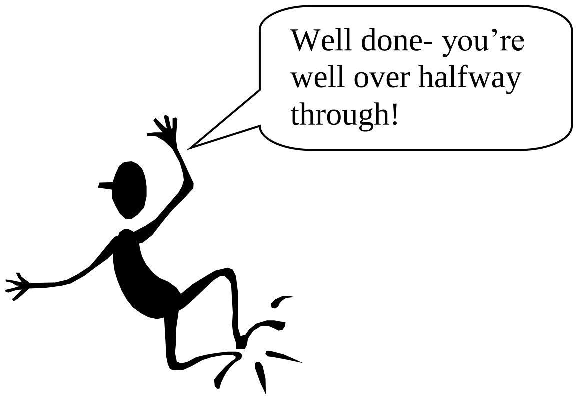

PLEASE CIRCLE ONE NUMBER FOR EACH QUESTION

These items are designed to measure beliefs and vivid mental experiences. We believe that many people have had some such experiences in their lives. Please answer the following questions as honestly as you can. *We are NOT interested in experiences people may have had under the influence of drugs*.

		NO	YES
**371.**	Do you ever feel as if people seem to drop hints about you or say things with a double meaning?	1	2
**372.**	Do you ever feel as if things in magazines or on TV were written especially for you?	1	2
**373.**	Do you ever feel as if some people are not what they seem to be?	1	2
**374.**	Do you ever feel as if you are being persecuted in some way?	1	2
**375.**	Do you ever feel as if there is a conspiracy against you?	1	2
**376.**	Do you ever feel as if you are or destined to be someone very important?	1	2
**377.**	Do you ever feel that you are a very special or unusual person?	1	2
**378.**	Do you ever feel that you are especially close to God?	1	2
**379.**	Do you ever think that people can communicate telepathically?	1	2
**380.**	Do you ever feel as if electrical devices such as computers can influence the way you think?	1	2
**381.**	Do you ever feel as if you have been chosen by God in some way?	1	2
**382.**	Do you believe in the power of witchcraft, voodoo or the occult?	1	2
**383.**	Are you often worried that your partner may be unfaithful?	1	2
**384.**	Do you ever feel that you have sinned more than the average person?	1	2
**385.**	Do you ever feel that people look at you oddly because of your appearance?	1	2
**386.**	Do you ever feel as if you had no thoughts in your head at all?	1	2
**387.**	Do you ever feel as if the world is about to end?	1	2
**388.**	Do your thoughts ever feel alien to you in some way?	1	2
**389.**	Have your thoughts ever been so vivid that you were worried other people would hear them?	1	2
**390.**	Do you ever feel as if your own thoughts were being echoed back to you?	1	2
**391.**	Do you ever feel as if you are a robot or zombie without a will of your own?	1	2

PLEASE CIRCLE ONE NUMBER FOR EACH QUESTION

**Show how much you agree with each of the following items:**		**Totally disagree**	**Strongly disagree**	**Slightly disagree**	**Slightly agree**	**Strongly agree**	**Totally agree**
**392.**	Overall, I am a worthwhile person	1	2	3	4	5	6
**393.**	I am easier to get to know than most people	1	2	3	4	5	6
**394.**	I feel confident that other people will be there for me when I need them	1	2	3	4	5	6
**395.**	I prefer to depend on myself rather than other people	1	2	3	4	5	6
**396.**	I prefer to keep to myself	1	2	3	4	5	6
**397.**	To ask for help is to admit that you're a failure	1	2	3	4	5	6
**398.**	People's worth should be judged by what they achieve	1	2	3	4	5	6
**399.**	Achieving things is more important than building relationships	1	2	3	4	5	6
**400.**	Doing your best is more important than getting on with others	1	2	3	4	5	6
**401.**	If you've got a job to do, you should do it no matter who gets hurt	1	2	3	4	5	6
**402.**	It's important to me that others like me	1	2	3	4	5	6
**403.**	It's important to me to avoid doing things that others won't like	1	2	3	4	5	6
**404.**	I find it hard to make a decision unless I know what other people think	1	2	3	4	5	6
**405.**	My relationships with others are generally superficial	1	2	3	4	5	6
**406.**	Sometimes I think I am no good at all	1	2	3	4	5	6
**407.**	I find it hard to trust other people	1	2	3	4	5	6
**408.**	I find that others are reluctant to get as close as I would like	1	2	3	4	5	6
**409.**	I find it relatively easy to get close to other people	1	2	3	4	5	6
**410.**	I find it easy to trust others	1	2	3	4	5	6
**411.**	I feel comfortable depending on other people	1	2	3	4	5	6
**412.**	I worry that others won't care about me as much as I care about them	1	2	3	4	5	6
**413.**	I worry about people getting too close	1	2	3	4	5	6
**414.**	I worry that I won't measure up to other people	1	2	3	4	5	6
**415.**	I have mixed feelings about being close to others	1	2	3	4	5	6
**416.**	While I want to get close to others I feel uneasy about it	1	2	3	4	5	6
**417.**	I wonder why people would want to be involved with me	1	2	3	4	5	6
**418.**	It's very important to me to have a close relationship	1	2	3	4	5	6

PLEASE CIRCLE ONE NUMBER FOR EACH QUESTION

Show how much you agree with each of the following items:		Totally disagree	Strongly disagree	Slightly disagree	Slightly agree	Strongly agree	Totally agree
**419.**	I worry a lot about my relationships	1	2	3	4	5	6
**420.**	I wonder how I would cope without someone to love me	1	2	3	4	5	6
**421.**	I feel confident about relating to others	1	2	3	4	5	6
**422.**	I often feel left out or alone	1	2	3	4	5	6
**423.**	I often worry that I do not really fit in with other people	1	2	3	4	5	6
**424.**	Other people have their own problems, so I don't bother them with mine	1	2	3	4	5	6
**425.**	When I talk over my problems with others, I generally feel ashamed or foolish	1	2	3	4	5	6
**426.**	I am too busy with other activities to put much time into relationships	1	2	3	4	5	6
**427.**	If something is bothering me, others are generally aware and concerned	1	2	3	4	5	6
**428.**	I am confident that other people will like and respect me	1	2	3	4	5	6
**429.**	I get frustrated when others are not available when I need them	1	2	3	4	5	6
**430.**	Other people often disappoint me	1	2	3	4	5	6

431. How often do you go to church?

Weekly…………………………… 1
Monthly…………………………… 2
Less than once a month……….. 3
Never…………………………….. 4

432. Do you believe in God?

No………………….. 1
Yes…………………. 2
Do not know………. 3

433. Do you believe in a spiritual or higher power other than God?

No………………….. 1
Yes…………………. 2
Do not know……… 3

434. How often do you participate in religious activities (eg. prayer, religious rituals)?

Never…………………………. 1
Less than once a month…… 2
Monthly………………………. 3
Weekly……………………….. 4
Daily………………………….. 5

PLEASE CIRCLE ONE NUMBER FOR EACH QUESTION

YOUR UPBRINGING

435. How many years did you live with your MOTHER up to age 16years?
(Write 0, if nil)

[ ][ ] years

436. Who was the person who MOST looked after you up to the age of 16 years?

Biological mother………………….. 1
Stepmother………………………… 2
Biological father…………………… 3
Stepfather………………………….. 4
Grandparent……………………….. 5
Other (please specify):
__________________________ 6

As you remember this person *(at question 436)* in your first 16 years, how much was she/he like the following?		Very like	Moderately like	Moderately unlike	Very unlike
**437.**	Spoke to me with a warm and friendly voice	1	2	3	4
**438.**	Did not help me as much as I needed	1	2	3	4
**439.**	Let me do those things I liked doing	1	2	3	4
**440.**	Seemed emotionally cold to me	1	2	3	4
**441.**	Appeared to understand my problems	1	2	3	4
**442.**	Was affectionate to me	1	2	3	4
**443.**	Liked me to make my own decisions	1	2	3	4
**444.**	Did not want me to grow up	1	2	3	4
**445.**	Tried to control everything I did	1	2	3	4
**446.**	Invaded my privacy	1	2	3	4
**447.**	Enjoyed talking things over with me	1	2	3	4
**448.**	Frequently smiled at me	1	2	3	4
**449.**	Tended to baby me	1	2	3	4
**450.**	Did not seem to understand what I needed or wanted	1	2	3	4
**451.**	Let me decide things for myself	1	2	3	4
**452.**	Made me feel I wasn't wanted	1	2	3	4
**453.**	Could make me feel better when I was upset	1	2	3	4
**454.**	Did not talk with me very much	1	2	3	4
**455.**	Tried to make me dependent on her/him	1	2	3	4
**456.**	Felt I could not look after myself unless she/he was around	1	2	3	4
**457.**	Gave me as much freedom as I wanted	1	2	3	4
**458.**	Let me go out as often as I wanted	1	2	3	4
**459.**	Was overprotective of me	1	2	3	4
**460.**	Did not praise me	1	2	3	4
**461.**	Let me dress any way I pleased	1	2	3	4

PLEASE CIRCLE ONE NUMBER FOR EACH QUESTION

SEXUAL HEALTH

462. At what age did you have sexual intercourse for the first time?
(Write 0 if never had sexual intercourse)

| | | years

463. How many sexual partners have you had in the LAST 12 MONTHS?

None…………………. 1
One…………………... 2
Two…………………... 3
Three………………… 4
Four………………….. 5
Five or more……….. 6

464. How old were you when you had your first period?
(Write 0 if you are male)

| | | years

465. Are you pregnant?
(Circle the NO option if you are male)

No………… 1
Yes……….. 2
Not sure…… 3

466. What sort of contraception do you use now?

Don't need any (pregnant, no periods, no sexual relationship)………….. 1
Choose not to use any……………………………………………………….. 2
Oral contraceptive pill………………………………………………………. 3
Condoms………………………………………………………………………. 4

Other *(Please specify)*____ 5

For the following questions, if you are FEMALE please answer the questions on the LEFT, and if MALE, please answer the questions on the RIGHT.
(Write 0 if nil)

FEMALES:

**How many times have you:**		**Number of times**
**467A.**	Been pregnant?	
**468A.**	Had a miscarriage?	
**469A.**	Had a termination?	
**470A.**	Given birth to a child?	

MALES:

		**Number**
**467B.**	How many pregnancies have you caused?	
**468B.**	How many of these pregnancies resulted in miscarriage?	
**469B.**	How many of these pregnancies resulted in termination/abortion?	
**470B.**	How many children have you fathered (biologically)?	

PLEASE CIRCLE ONE NUMBER FOR EACH QUESTION

PLEASE NOTE THAT YOUR ANSWERS TO THE FOLLOWING QUESTIONS WILL BE KEPT IN STRICTEST CONFIDENCE.

If in answering these questions you feel distressed and wish to discuss the matter, please refer to the contact details of available counselling services *(attached to the back of this questionnaire).*

PERSONAL

471. Have you EVER been pressured or forced to have sexual contact when you did not want to?

No……………………………………………….. 1
Unsure….…………………………………….… 2
Yes……………………………………………..….. 3

472. How many times did you have this experience BEFORE you were 16?
(Write 0, if nil)

[ ] Times

473. How many times did you have this experience AFTER the age of 16?
(Write 0, if nil)

[ ] times

Did ANY of the following events happen to you BEFORE AGE 16?		No	Unsure	Yes
**474.**	Someone exposed themselves or masturbated in front of you	1	2	3
**475.**	Someone more than 5 years older than you kissed or fondled your breasts or genitals	1	2	3
**476.**	Someone more than 5 years older than you had sexual intercourse with you	1	2	3
**477.**	Someone more than 5 years older than you had oral sex with you	1	2	3
**478.**	You touched or masturbated the genitals of someone more than 5 years older than you	1	2	3

479. Were you able to tell anyone about these experiences?

Experiences didn't happen….. 1
No……………………………… 2
Yes…………………………….... 3

480. If you were able to tell, who did you tell?

Experiences didn't happen/didn't tell ………… 1
Mother………………………………………………. 2
Father………………………………………………. 3
Other family member………………………………. 4
Someone outside the family……………………… 5

PLEASE CIRCLE ONE NUMBER FOR EACH QUESTION

481. If you were able to tell someone about these experiences, did you feel that the person you told believed you?

No experiences/didn't tell……………………………..	1
Not at all believed…………………………………….	2
Somewhat believed…………………………………..	3
Believed……………………………………………….	4
Totally believed me…………………………………..	5

Thinking back on this experience, how much do you agree that the following are to blame for your experience?	Didn't happen	Strongly Disagree	Slightly disagree	Neither agree or disagree	Slightly agree	Strongly Agree
**482.** Society in general	1	2	3	4	5	6
**483.** The person(s) who did this to me	1	2	3	4	5	6
**484.** My behaviour (how I act)	1	2	3	4	5	6
**485.** My character (who I am)	1	2	3	4	5	6
**486.** Other	1	2	3	4	5	6

487. Have you EVER been raped (forced to have sexual intercourse)?

No……………………….	1
Once……………………	2
Twice or more times…..	3

488. If you have been raped, how old were you the first time you were raped?
(*Write 0 if this question does not apply to you*)

[ ] years

489. Did you report this event to the police?

No event to report……………..	1
No, I didn't report the event…..	2
Yes………………………………	3

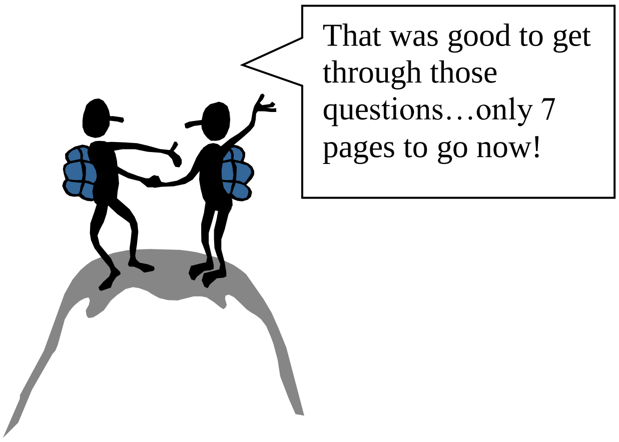

PLEASE CIRCLE ONE NUMBER FOR EACH QUESTION

How OFTEN have any of the following EVER happened in your relationship with a partner *(in either current or previous relationships)*?

(*Circle* **No partner** *if you've never had a partner)*

My partner:		No partner	Never	Only once	Several times	Once per month	Once per week	Daily
**490.**	Told me that I wasn't good enough	1	2	3	4	5	6	7
**491.**	Harassed me by following me	1	2	3	4	5	6	7
**492.**	Tried to turn my family, friends and/or children against me	1	2	3	4	5	6	7
**493.**	Slapped me	1	2	3	4	5	6	7
**494.**	Tried to make me think I was crazy	1	2	3	4	5	6	7
**495.**	Accused me of having an affair when I wasn't	1	2	3	4	5	6	7
**496.**	Threatened to hit me or throw something at me	1	2	3	4	5	6	7
**497.**	Raped me	1	2	3	4	5	6	7
**498.**	Blamed me for causing his/her violent behaviour	1	2	3	4	5	6	7
**499.**	Harassed me over the telephone	1	2	3	4	5	6	7
**500.**	Harassed me at work	1	2	3	4	5	6	7
**501.**	Tried to keep me from seeing or talking to my family	1	2	3	4	5	6	7
**502.**	Pushed, shoved or grabbed me	1	2	3	4	5	6	7
**503.**	Became upset if dinner/housework wasn't done when they thought it should be	1	2	3	4	5	6	7
**504.**	Told me that I was crazy	1	2	3	4	5	6	7
**505.**	Told me that no one would ever want me	1	2	3	4	5	6	7
**506.**	Did not want me to socialise with my same-sex friends	1	2	3	4	5	6	7
**507.**	Tried to convince my friends, family or children that I was crazy	1	2	3	4	5	6	7
**508.**	Told me that I was stupid	1	2	3	4	5	6	7
**509.**	Hit or tried to hit me with something	1	2	3	4	5	6	7
**510.**	Harassed me by hanging around outside my house	1	2	3	4	5	6	7
**511.**	Told me that I was ugly	1	2	3	4	5	6	7
**512.**	Kicked me, bit me or hit me with a fist	1	2	3	4	5	6	7
**513.**	Forced me to have sex with other partners	1	2	3	4	5	6	7
**514.**	Takes/took control of all decision making	1	2	3	4	5	6	7

PLEASE CIRCLE ONE NUMBER FOR EACH QUESTION

**My partner:** (*Circle* **No partner** *if you've never had a partner)*		No partner	Never	Only once	Several times	Once per month	Once per week	Daily
**515.**	Wants/wanted me to do all the work around the house	1	2	3	4	5	6	7
**516.**	Takes no interest/wasn't interested in what I do	1	2	3	4	5	6	7
**517.**	Doesn't/didn't show me affection	1	2	3	4	5	6	7
**518.**	Sulks/sulked and refuses/refused to talk to me	1	2	3	4	5	6	7
**519.**	Used a knife or gun or other weapon	1	2	3	4	5	6	7

ACTIVITIES AND INTERESTS

**In the LAST WEEK, how many hours in total did you spend on the computer for the following purposes?** *(Write 0 if no time spent)*		Number of hours	
**520.**	Work		
**521.**	Recreation		
**522.**	Study		

523. **In the PAST WEEK, how many hours in total did you spend on the internet for recreational purposes (*such as chat-rooms, surfing the net, e-mails, multiplayer games*)?**

[ ][ ] Hours

524. **On average, how many HOURS A DAY (Monday to Friday) do you sit and watch TV?**

7 hours or more…………… 1
5 to less than 7 hours…… 2
3 to less than 5 hours…… 3
1 to less than 3 hours…… 4
Less than 1 hour………….. 5
Never watch TV…………… 6

525. **On average, how many HOURS A DAY (Saturday and Sunday) do you sit and watch TV?**

7 hours or more…………… 1
5 to less than 7 hours…… 2
3 to less than 5 hours…… 3
1 to less than 3 hours…… 4
Less than 1 hour………….. 5
Never watch TV…………… 6

PLEASE CIRCLE ONE NUMBER FOR EACH QUESTION

526. Can you play any musical instruments?

No…………………………………… 1

Yes *(please name)*: 2

____

____

527. If you do play a musical instrument/s, how many HOURS PER WEEK do you generally spend on this activity?

(Write 0 if no instrument/s played or don't currently play)

[ ] hours per week

528. During the PAST 12 MONTHS, have you been involved in any creative pursuits or hobbies *(pottery, sewing, drawing, painting, woodwork, metalwork, dancing, acting, songwriting, etc)?*

No…………………………………… 1

Yes *(please name):* 2

____

____

529. Have you EVER involved yourself with any environmental activities *(such as being a member of an environmental group, or involving yourself in tree planting, land clean-up operations, or the protection of animal or plant species)*?

No………………………………… 1

Yes *(please name):* 2

____

____

530. Have you EVER been involved in any activities organised by community groups to improve community health and/or well-being *(eg. participation in fundraising activities such as 'fun-runs' or walkathons, community group membership, volunteer work)*?

No………………………………… 1

Yes *(Please specify):* 2

____

____

531. Have you EVER received any awards as a result of your activities within the community or particular fields of interest?

No………………………………… 1

Yes *(Please name)*: 2

____

____

YOUR SIZE

532. **How tall are you without shoes** *(see driver's licence for correct height)***?**

________ cms

533. **How much do you weigh in light clothes without shoes?** ________ kgs

For the following measurements, please remove heavy outer garments and remove contents of pockets.

534. **What is your waist measurement?** ________ cms
(measure around waist at navel level)

535. **What is your hip measurement?** ________ cms
(measure around hips at point of greatest width)

536. **How much would you like to weigh?**

I'd like to be more than 10kg lighter......... 1
I'd like to be 6 – 10kg lighter.................. 2
I'd like to be 1-5 kg lighter..................... 3
I'm happy as I am.............................. 4
I'd like to be 1 – 5 kg heavier................ 5
I'd like to be more than 5kg heavier 6

537. **How often have you gone on a diet to LOSE WEIGHT during the last year?**

Never.. 1
1 – 4 times.. 2
5 – 10 times.. 3
More than 10 times............................... 4
I am always on a diet to lose weight.......... 5

538. **How would you describe yourself now?**

Very underweight................................. 1
Underweight... 2
Slightly underweight.............................. 3
Average... 4
Slightly overweight............................... 5
Overweight... 6
Very overweight................................... 7

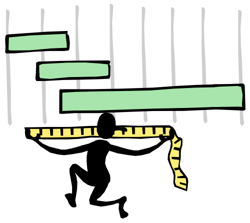

PLEASE CIRCLE ONE NUMBER FOR EACH QUESTION

GAMBLING BEHAVIOUR

The following questions are about activities you may participate in (whether at a club, casino, hall, hotel, at home or other place) and include the following:

- ***playing poker or gaming machines, keno, table games such as blackjack or roulette, bingo, casino games on the internet, games like cards or mahjong for money***
- ***betting on horse or greyhound races, or sporting events (excluding sweeps)***
- ***buying instant scratch tickets, lotto or any other lottery game like Gold Lotto, Powerball, OZ Lotto, the Pools or bought lottery tickets, raffle or Art Union tickets***
- ***any other gambling activity***

**In the LAST 12 MONTHS:**		**Never**	**Rarely**	**Sometimes**	**Often**	**Always**	**Don't know/ Can't remember**
**539.**	Have you bet more than you could really afford to lose?	1	2	3	4	5	6
**540.**	Have you needed to gamble with larger amounts of money to get the same feeling of excitement?	1	2	3	4	5	6
**541.**	When you gambled, did you go back another day to try to win back the money you lost?	1	2	3	4	5	6
**542.**	Have you borrowed money or sold anything to get money to gamble?	1	2	3	4	5	6
**543.**	Have you felt that you might have a problem with gambling?	1	2	3	4	5	6
**544.**	Has gambling caused you any health problems, including stress or anxiety?	1	2	3	4	5	6
**545.**	Have people criticized your betting or told you that you had a gambling problem, regardless of whether or not you thought it was true?	1	2	3	4	5	6
**546.**	Has your gambling caused any financial problems for you or your household?	1	2	3	4	5	6
**547.**	Have you felt guilty about the way you gamble or what happens when you gamble?	1	2	3	4	5	6
**548.**	Have you lied to family members or others to hide your gambling?	1	2	3	4	5	6
**549.**	Have you bet or spent more money than you wanted to on gambling?	1	2	3	4	5	6
**550.**	Have you wanted to stop betting money or gambling, but didn't think you could?	1	2	3	4	5	6

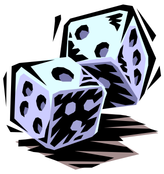

PLEASE CIRCLE ONE NUMBER FOR EACH QUESTION

The following questions are about your OPINIONS about gambling, as well as any EARLY EXPERIENCES you have had with gambling or betting money.

**Would you say you strongly agree, agree, disagree or strongly disagree with the following statements?**		**Strongly agree**	**Agree**	**Disagree**	**Strongly disagree**	**Don't know**
**551.**	After losing many times in a row, you are more likely to win.	1	2	3	4	5
**552.**	You could win more if you used a certain system or strategy.	1	2	3	4	5

553. Do you remember a big win, when you FIRST STARTED gambling?

Yes……………………………………. 1
No……………………………………... 2
Never gambled……………………… 3
Don't know/can't remember……….. 4

554. Do you remember a big loss, when you FIRST STARTED gambling?

Yes……………………………………. 1
No……………………………………... 2
Never gambled……………………… 3
Don't know/can't remember……….. 4

555. Has anyone in your immediate family EVER had a gambling problem?

Yes……………………………………. 1
No……………………………………... 2
Don't know/can't remember……….. 3

556. Has anyone in your immediate family EVER had an alcohol or a legal or illegal drug problem?

Yes……………………………………. 1
No……………………………………... 2
Don't know/can't remember……….. 3

**In the LAST 12 MONTHS, have you:**		**Yes**	**No**	**Don't know/ Can't remember**
**557.**	Used alcohol or drugs while gambling?	1	2	3
**558.**	Gambled while under the influence of alcohol or legal or illegal drugs?	1	2	3
**559.**	Felt you might have an alcohol or drug problem?	1	2	3

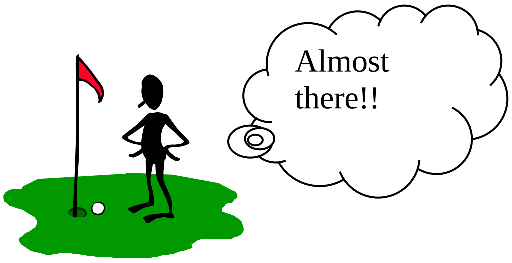

PLEASE CIRCLE ONE NUMBER FOR EACH QUESTION

**In the LAST 12 MONTHS:**		**Yes**	**No**	**Don't know/ Can't remember**
**560.**	If something painful happened in your life, did you have the urge to gamble?	1	2	3
**561.**	If something painful happened in your life, did you have the urge to have a drink?	1	2	3
**562.**	If something painful happened in your life, did you have the urge to use drugs or medication?	1	2	3
**563.**	Have you been under a doctor's care because of physical or emotional problems brought on by stress?	1	2	3
**564.**	Have you felt seriously depressed?	1	2	3

565. Has your gambling EVER adversely affected how well you perform in your job?

Never………………………………………………………… 1
Rarely ……………………………………………………… 2
Sometimes…………………………………………………… 3
Often………………………………………………………… 4
Always ……………………………………………………… 5
Don't know/can't remember………………………………… 6

566. Has your gambling EVER left you with not enough time to look after your family's interests?

Never………………………………………………………… 1
Rarely ……………………………………………………… 2
Sometimes…………………………………………………… 3
Often………………………………………………………… 4
Always ……………………………………………………… 5
Don't know/can't remember………………………………… 6

		**Yes**	**No**	**Don't know/ Can't remember**
**567.**	Have you EVER changed jobs because of problems relating to your gambling?	1	2	3
**568.**	Have you EVER been sacked from a job because of your gambling?	1	2	3
**569.**	Has your gambling EVER led you to obtain money illegally, even if you intended to pay it back?	1	2	3
**570.**	Have you EVER been in trouble with the police because of activities related to your gambling?	1	2	3

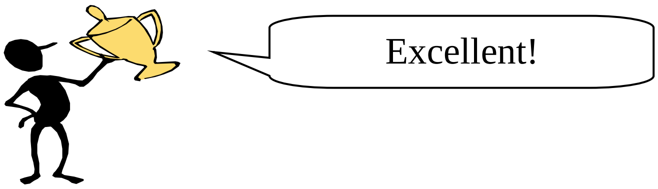

Thank you for your participation and assistance. If you would like to make any comments, please write these on the back of this page.
