# Supplementary material for: Effect of maternal smoking in pregnancy and childhood on child and adolescent sleep outcomes to 21 years: a birth cohort study
Source: BMC Pediatr. 2019 Mar 6;19:70. doi: 10.1186/s12887-019-1439-1 (PMC6402153; doi:10.1186/s12887-019-1439-1)
Supplement: Supplementary file 9 — MUSP smoking and sleep input data codebook. (PDF 354 kb) [file 12887_2019_1439_MOESM9_ESM.pdf]

Code book

At first-antenatal visit

Ragem variable level "age mother at fcv"
Value level
1 "13-19"
2 "20-35"
3 "35+"
Ra90 variable level "recorded income at phase A"
Value level
1 "$10400 or more"
2 "$10399 or less"

Ra80 variable level " Maternal education at FCV"
Value level
1 " incomplete high "
2 " complete high "
3 " post high "
Rbabwant variable level "pregnancy planned-wanted"
Value level
1 " plan-want"
2 " unsure "
3 "unplan-want"

a68 variable level "HOW MANY CIGARETTE DAILY: PRE-PREG"
Value label
1 " 50 OR MORE"
2 "30 - 49 PER DAY"
3 "20 - 29 PER DAY"
4 "10-19 PER DAY"
5 " 1-9 PER DAY"
6 "NONE"

a73 variable level "HOW MANY CIGARETTES DAILY: LAST WEEK"
Value label
1 " 50 OR MORE"
2 "30 - 49 PER DAY"
3 "20 - 29 PER DAY"
4 "10-19 PER DAY"
5 " 1-9 PER DAY"
6 "NONE"

a77 variable level " CUPS COFFEE DAILY: SINCE PREG"
value level
1 " 7 CUPS OR MORE"
2 "4-6 CUPS PER DAY"
3 "2-3 CUPS PER DAY"
4 " 1 CUP PER DAY"

5 “NONE”

a78 variable level “ CUPS TEA DAILY: SINCE PREG”
value level
6 “ 7 CUPS OR MORE”
7 “4-6 CUPS PER DAY”
8 “2-3 CUPS PER DAY”
9 “ 1 CUP PER DAY”
10 “NONE”
ralc3 variable level “mat alcohol late”
value level
1 “ nil”
2 “>0 - .5 per day “
3 “>.5 - 1 per day “
4 “ >1 per day “

At birth

b35 variable level “ HOW MANY CIGARETTES DAILY - LAST 3 MTHS – PHB”
Value label
1 “ 50 OR MORE”
2 “30 - 49 PER DAY”
3 “20 - 29 PER DAY”
4 “10-19 PER DAY”
5 “ 1-9 PER DAY”
6 “NONE”

b38 variable level “ CUPS COFFEE DAILY: SINCE PREG”
value level
11 “ 7 CUPS OR MORE”
12 “4-6 CUPS PER DAY”
13 “2-3 CUPS PER DAY”
14 “ 1 CUP PER DAY”
15 “NONE”

b39 variable level “ CUPS TEA DAILY: SINCE PREG”
value level
16 “ 7 CUPS OR MORE”
17 “4-6 CUPS PER DAY”
18 “2-3 CUPS PER DAY”
19 “ 1 CUP PER DAY”
20 “NONE”

6-month

e75 variable level “ LAST WEEK HOW MANY CIGARETTES SMOKE PER DAY”
Value label
1 “ 50 OR MORE”
2 “30 - 49 PER DAY”
3 “20 - 29 PER DAY”

4 “10-19 PER DAY”
5 “ 1-9 PER DAY”
6 “NONE”

e2 variable level “sleeplessness”
Value level
1 “almost every day”
2 “ few times a week”
3 “few times a month”
4 “ rarely”

E17 variable level “how long did you beast feed”
Value level
1 “still breast feeding “
2 “ 4-6 months “
3 “ 7 wks to 3 mths “
4 “ 3 wks to 6 wks “
5 “ 2 wks or less “
6 “ not at all “

5-years
h93 variable level “IN LAST WEEK HOW MANY CIGARETTES PER DAY”
Value label
1 “ 50 OR MORE”
2 “30 - 49 PER DAY”
3 “20 - 29 PER DAY”
4 “10-19 PER DAY”
5 “ 1-9 PER DAY”
6 “NONE”

g96 variable level “trouble sleeping”
Value level
1 “often”
2 “sometimes”
3 “never”

14-years
**variable name with “k” reported by mothers and with “j” reported by child*

k196 variable level “CIGS PER DAY IN LAST WEEK”
Value label
1 “ 50 OR MORE”
2 “30 - 49 PER DAY”
3 “20 - 29 PER DAY”
4 “10-19 PER DAY”
5 “ 1-9 PER DAY”
6 “NONE”

k47 variable level “nightmares”
Value level
1 “often”

2 “sometimes”
3 “never”
k54 variable level “overtired”
Value level
1 “often”
2 “sometimes”
3 “never”
k76 variable level “sleeps more than most kids day or night”
Value level
1 “often”
2 “sometimes”
3 “never”
k91 variable level “ talks or walks in sleep”
Value level
1 “often”
2 “sometimes”
3 “never”
k99 variable level “trouble sleeping”
Value level
1 “often”
2 “sometimes”
3 “never”
k150 variable level “child-snoring in last year”
Value level
1 “often”
2 “sometimes”
3 “never”
k75 variable level “ sleeps less than most kids”
Value level
1 “often”
2 “sometimes”
3 “never”
j47 variable level “nightmares”
Value level
1 “often”
2 “sometimes”
3 “never”

j54 variable level “feel overtired”
Value level
1 “often”
2 “sometimes”
3 “never”
j76 variable level “sleep more than most kids”
Value level
1 “often”
2 “sometimes”
3 “never”
j91 variable level “talks or walks in sleep”
Value level

1 “often”
2 “sometimes”
3 “never”
j99 variable level “ trouble sleeping”
Value level
1 “often”
2 “sometimes”
3 “never”
j75 variable level “sleep less than most kids”
Value level
1 “often”
2 “sometimes”
3 “never”

21-years

m319 variable level “trouble sleeping”
Value level
1 “not true”
2 “somewhat-sometimes true”
3 “very-often true”
m334 variable level “ have nightmares”
Value level
1 “not true”
2 “somewhat-sometimes true”
3 “very-often true”
m274 variable level “ feel overtired”
Value level
1 “not true”
2 “somewhat-sometimes true”
3 “very-often true”
m215 variable level “restlessness sleep”
Value level
1 “ < once a week “
2 “once to twice per week”
3 “three+ per week”
m213 variable level “ waking during night”
Value level
1 “ < once a week “
2 “once to twice per week”
3 “three+ per week”
m216 variable level “ daytime drowsiness”
Value level
1 “ < once a week “
2 “once to twice per week”
3 “three+ per week”
m219 variable level “ trouble staying awake”
Value level
1 “ no “
2 “<once a week”
3 “once or twice week”
4 “three +per week”

m217 variable level "sleep quality"
Value level
1 "very good"
2 "fairly good"
3 "fairly bad"
4 "very bad"
m214 variable level " snoring"
Value level
1 "<once a week"
2 "once or twice week"
3 "three +per week"
